# Sub-Inhibitory Concentrations of Human α-defensin Potentiate Neutralizing Antibodies against HIV-1 gp41 Pre-Hairpin Intermediates in the Presence of Serum

**DOI:** 10.1371/journal.ppat.1003431

**Published:** 2013-06-13

**Authors:** Lusine Demirkhanyan, Mariana Marin, Wuyuan Lu, Gregory B. Melikyan

**Affiliations:** 1 Division of Pediatric Infectious Diseases, Emory University Children's Center, Atlanta, Georgia, United States of America; 2 Institute of Human Virology and Department of Biochemistry, University of Maryland School of Medicine, Baltimore, Maryland, United States of America; 3 Children's Healthcare of Atlanta, Atlanta, Georgia, United States of America; University of Zurich, Switzerland

## Abstract

Human defensins are at the forefront of the host responses to HIV and other pathogens in mucosal tissues. However, their ability to inactivate HIV in the bloodstream has been questioned due to the antagonistic effect of serum. In this study, we have examined the effect of sub-inhibitory concentrations of human α-defensin HNP-1 on the kinetics of early steps of fusion between HIV-1 and target cells in the presence of serum. Direct measurements of HIV-cell fusion using an enzymatic assay revealed that, in spite of the modest effect on the extent of fusion, HNP-1 prolonged the exposure of functionally important transitional epitopes of HIV-1 gp41 on the cell surface. The increased lifetime of gp41 intermediates in the presence of defensin was caused by a delay in the post-coreceptor binding steps of HIV-1 entry that correlated with the marked enhancement of the virus' sensitivity to neutralizing anti-gp41 antibodies. By contrast, the activity of antibodies to gp120 was not affected. HNP-1 appeared to specifically potentiate antibodies and peptides targeting the first heptad repeat domain of gp41, while its effect on inhibitors and antibodies to other gp41 domains was less prominent. Sub-inhibitory concentrations of HNP-1 also promoted inhibition of HIV-1 entry into peripheral blood mononuclear cells by antibodies and, more importantly, by HIV-1 immune serum. Our findings demonstrate that: (i) sub-inhibitory doses of HNP-1 potently enhance the activity of a number of anti-gp41 antibodies and peptide inhibitors, apparently by prolonging the lifetime of gp41 intermediates; and (ii) the efficiency of HIV-1 fusion inhibitors and neutralizing antibodies is kinetically restricted. This study thus reveals an important role of α-defensin in enhancing adaptive immune responses to HIV-1 infection and suggests future strategies to augment these responses.

## Author Summary

Human neutrophil peptide 1 (HNP-1) is a small cationic peptide that can directly block HIV-1 entry in the absence of serum. However, since serum attenuates the anti-HIV activity of this peptide, HNP-1 is unlikely to inhibit infection in the bloodstream. Here, we demonstrate that sub-inhibitory doses of HNP-1 in the presence of serum can strongly enhance the activity of neutralizing antibodies and inhibitors targeting transiently exposed intermediate conformations of HIV-1 gp41. HNP-1 appears to exert this effect by delaying post-coreceptor binding steps of fusion and thereby prolonging the exposure of gp41 intermediates. These results imply that the HIV-1 fusion kinetics is an important determinant of sensitivity to neutralizing antibodies and peptides against transiently exposed functional domains of gp41. The surprising synergy between sub-inhibitory concentrations of HNP-1 and anti-gp41 antibodies suggests new strategies to sensitize the virus to circulating antibodies by developing compounds that prolong the exposure of conserved gp41 epitopes on the cell surface.

## Introduction

HIV envelope glycoprotein (Env) is a trimer each monomer of which consists of non-covalently associated transmembrane (gp41) and surface (gp120) subunits [1]. The gp120 binding to CD4 induces the formation of the gp120 coreceptor binding site and enables recruitment of HIV coreceptors (CCR5 or CXCR4) [2], [3]. The formation of ternary Env-CD4-coreceptor complexes triggers the gp41 refolding into the final 6-helix bundle (6HB) structure [4], [5]. In the 6HB structure, the three C-terminal heptad repeat (C-HR) domains bind in an antiparallel orientation to the hydrophobic grooves of the central N-terminal heptad repeat domain (N-HR). The refolding of gp41 from a native conformation to the 6HB is a multi-step process that proceeds through several intermediate conformations which expose N-HR and C-HR domains [5], [6] and are collectively referred to as pre-bundle or pre-hairpin intermediates (PHIs).

Synthetic peptides derived from the N-HR and C-HR regions inhibit HIV-1 fusion by binding to complementary domains on the gp41 PHIs and blocking the 6HB formation [4]. The gp41 N-HR and C-HR domains are exposed/formed after binding to CD4 or coreceptors, but are not available on the native Env or on the final 6HB structure [7]–[9]. Hence, the inhibitory peptides have a limited window of opportunity to bind to gp41 and block HIV-1 fusion. Functional evidence implies that the time of PHI exposure is a major determinant of the potency of C-HR-derived peptides [10]–[13]. Specifically, the rate of fusion/infection has been shown to correlate with the HIV-1 resistance to neutralizing antibodies and C-HR-derived peptides [10], [14]. The lifetime of PHIs on the cell surface is a function of the relative rates of their formation upon CD4 binding and disappearance, which could be due to: (i) conversion to 6HBs upon virus fusion with the plasma membrane and/or (ii) virus clearance from the cell surface followed by fusion with endosomes [15]. The former pathway is operational in a cell-cell fusion model (e.g., [8], [16]), whereas the second mechanism appears to be responsible for the HIV-1 escape from peptide inhibitors [10], [15] (see below).

In addition to the relatively short lifetime of PHIs in the course of fusion, HIV-1 entry *via* endocytosis reveals a novel escape pathway from the peptide inhibitors [15]. Quick HIV-1 uptake following the interactions with CD4 and coreceptors would limit the cell surface exposure of PHIs and thus increase the virus resistance to inhibitors targeting intermediate conformations of Env. Indeed, the inhibitory potency of C-HR-derived peptides is enhanced upon imposing a transient block on HIV-1 endocytosis [15], [17]. We therefore hypothesized that the sensitivity of HIV-1 to neutralizing antibodies against transiently exposed Env epitopes is modulated by the lifetime of surface-accessible PHIs [10]. This notion is supported by the synergy between a gp41-derived peptide that appears to stabilize PHI and anti-gp41 antibodies [14]. Thus, in addition to steric restrictions on antibody binding [18]–[23], kinetic factors (such as the lifetime of PHIs and the on/off rates of antibody binding) may contribute to the ability of antibodies to engage transiently exposed epitopes [12], [24], [25]. For example, antibodies against CD4-induced epitopes neutralize HIV-1 more potently in cells expressing low levels of coreceptors or in the presence of coreceptor antagonists [13], [26]–[28]; these conditions are known to slow down HIV-1 fusion [10], [13].

The above considerations suggest that the rate of HIV-1 uptake/fusion can modulate the virus' resistance to entry inhibitors. Our recent study revealed that human α-defensin HNP-1 interferes with several steps of HIV-1 fusion and also selectively inhibits productive uptake of this virus [29]. We therefore asked whether low concentrations of HNP-1 can enhance the activity of antibodies and fusion inhibitors targeting intermediate conformations of Env by delaying the HIV-1 uptake and/or fusion. By examining the effect of HNP-1 on the kinetics of virus fusion, we confirmed that even marginally inhibitory doses of defensin in the presence of human serum increased the lifetime of PHIs on the cell surface. The longer exposure of gp41 intermediates correlated with dramatic potentiation of the inhibitory activity of antibodies and peptides targeting the N-HR domain. By comparison, a less marked enhancement of antiviral activities by defensin was observed for peptides and antibodies targeting other gp41 domains, while HIV-1 neutralization by anti-gp120 antibodies was not affected under our conditions. Importantly, the strong enhancing effect on HIV-1 neutralization was observed in the presence of serum, which antagonizes the ability of HNP-1 to directly inhibit fusion [29]–[32]. Our results thus demonstrate a remarkable synergy between innate and adaptive immune responses in blocking HIV-1 entry and fusion.

## Results

### Kinetically resolved intermediates of HIV-cell fusion

**Figure 1:**
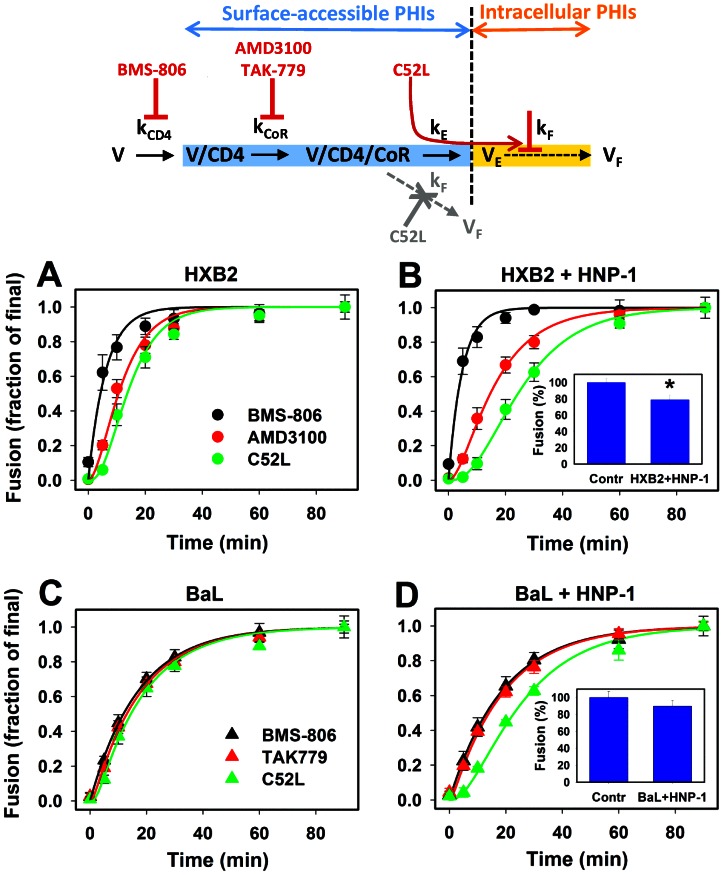
HNP-1 stabilizes HIV-1 pre-hairpin intermediates. *Top*: A kinetic model of HIV-cell fusion. Progression of virus fusion through the surface accessible steps – receptor binding (V/CD4), coreceptor binding (V/CD4/CoR) and endocytosis (V_E_) – is measured by adding specific inhibitors at different time intervals. Here, k_CD4_, k_CoR_ and k_E_ are the effective rate constants (k_F_ is the rate constant for HIV-endosome fusion (V_F_) that is not resolved by the current approach). The steps that form/expose pre-hairpin intermediates (PHI) on the cell surface are highlighted in blue, while intracellular PHIs, which are no longer accessible to added inhibitors, are highlighted in orange (a vertical dashed line separates internalized viruses from external viruses). C52L is carried over by the PHIs into endosomes (curved red arrow) where it blocks subsequent fusion. The virus is assumed to inactivate at every step of the fusion reaction through detachment from cells and by undergoing non-productive endocytosis (not shown for visual clarity). The inactivation rate constant k_i_ was estimated based on the rate of non-productive endocytosis measured in [10]. An alternative pathway for HIV-1 escape from C52L by fusing with the plasma membrane is shown by a dashed arrow and is colored gray. In this case, the kinetics of the V/CD4/CoR escape from C52L is described by k_F_. (A–D) HXB2 pseudoviruses (A, B) or BaL pseudoviruses (C, D) were pre-bound to TZM-bl cells in the cold and allowed to undergo fusion for 90 min at 37°C, either in the absence (A, C) or in the presence (B, D) of 7.3 µM HNP-1 in HBSS supplemented with 10% human serum. At indicated time points, fully inhibitory concentrations of HIV-1 fusion inhibitors, BMS-806, AMD3100, TAK-779 or C52L, were added, and incubation was continued till the 90 min point. The resulting virus fusion was measured by the BlaM assay, as described in Materials and Methods. Data points are means and SEM from 2 independent experiments performed in triplicate. Solid lines are obtained by curve-fitting using the three-step kinetic model (see also [10]). The calculated effective rate constants are given in Table 1. *Insets* in panels B and D show the effect of 7.3 µM HNP-1 in serum-containing medium on HXB2 and BaL fusion, respectively. *, P<0.04 (two-tailed t-test).

We and others have shown that the ability of HNP-1 to inhibit HIV-1 fusion/infection is markedly attenuated in the presence of serum [29], [30]. Surprisingly, however, serum does not interfere with the HNP-1 binding to cellular and viral targets [29], implying that the binding itself does not confer anti-viral activity. Here, we asked whether, in spite of its poor inhibitory activity in the presence of serum, the bound HNP-1 can modulate the kinetics of HIV-1 fusion and thus enhance the potency of inhibitors and neutralizing antibodies. The kinetics of HIV-1 fusion and the longevity of PHIs have been measured by adding specific inhibitors of HIV-1 fusion at varied times of virus-cell incubation [10], [15], [17], [29]. From these data, the average lifetime of PHIs can be estimated using a simple three-step kinetic model (Fig. 1 and [10]). This approach revealed correlation between the longevity of PHIs and the inhibitory activity of C-HR-derived peptides.

According to our kinetic model, HIV-1, which is initially attached to a target cell in the cold through non-specific interactions, proceeds through the following three surface-accessible steps of fusion (Fig. 1): binding to CD4 (denoted V/CD4) and coreceptors (V/CD4/CoR) followed by productive endocytosis (V_E_). The virus progression through sequential steps of fusion is assessed by adding high concentrations of specific fusion inhibitors at different time points. This approach is insensitive to reversible engagement of receptor/coreceptor and thus measures the effective rate constants of progression beyond the fusion steps that are dependent on respective cellular proteins. The internalization of ternary HIV-CD4-CoR complexes culminates in fusion with endosomes (V_F_) [15], but this step is not kinetically resolved through addition of membrane-impermeant inhibitors and therefore does not contribute to the measured rate constants. We have obtained evidence for the existence of this post-endocytosis fusion step through arresting it at low temperature [15].

The acquisition of resistance to gp41-derived peptide inhibitors has been traditionally interpreted as HIV-1 fusion (e.g., [8], [33]). However, the finding that HIV-1 enters through endocytosis [15] suggests that kinetics of escape from these peptides reflects the rate of productive endocytosis which protects the virus from inhibitors and culminates in subsequent fusion with endosomes (Fig. 1). Importantly, the longevity of surface-accessible PHIs measured by our approach is independent of whether HIV-1 escapes from the C-HR-derived peptides by forming the gp41 6HBs, which implies direct fusion with the plasma membrane (Fig. 1, gray dashed arrow), or through productive endocytosis [10].

### Human α-defensin slows down Env-CXCR4 binding and productive HIV-1 endocytosis

To synchronize the HIV-1 fusion reaction, viruses were pre-bound to target cells in the cold, and virus entry was initiated by shifting to 37°C. Fully inhibitory concentrations of BMS-806 (blocks CD4-induced conformational changes [34], [35]), AMD3100 or TAK-779 (block CXCR4 and CCR5 binding, respectively [36], [37]) or the gp41-derived peptide C52L (prevents gp41 refolding [38]) were added at varied times of incubation. In agreement with our previous kinetic studies [10], HXB2 acquired resistance to the CD4 binding inhibitor ∼3-fold faster than BaL (Fig. 1A, C). Subsequent steps of CXCR4 binding and acquisition of resistance to C52L by HXB2 occurred after significant delays, whereas BaL engaged CCR5 and escaped the C52L inhibition shortly after binding CD4. Accordingly, the rate constants calculated based on the three-step kinetic model of HIV-1 fusion (Fig. 1 and [10]) reflected the slow progression of the post-CD4 binding steps of HXB2 fusion compared to BaL fusion (Table 1). From the derived rate constants, we estimated the average lifetime of PHIs defined as the combined time the Env exists in CD4- and CD4/CoR-bound states (for details, please see [10]). Our estimate showed that the HXB2 and BaL PHIs were exposed to inhibitory peptides for ∼6 min and ∼0.8 min, respectively. The much longer lifetime of HXB2 intermediates is consistent with the higher sensitivity of this virus to gp41-derived inhibitory peptides (Table 2 and [10]).

**Table 1:** Effect of HNP-1 on the kinetics of HIV-1 entry.

Virus	k_CD4_ (min^−1^)		k_CoR_ (min^−1^)**		k_E_/k_F_ (min^−1^)**	
	Control	HNP-1	Control	HNP-1	Control	HNP-1
**HXB2**	0.111±0.022*	0.157±0.023	0.102±0.019	0.0221±0.003	0.288±0.089	0.0521±0.007
**BaL**	0.029±0.001	0.023±0.002	0.855±0.244	1.071±0.434	0.685±0.285	0.091±0.013

k_CD4_, is the rate constant for CD4 binding; k_CoR_, is the rate constant for coreceptor binding; k_E_, is the rate constant for productive endocytosis, which is equivalent to k_F_ in the alternative kinetic scheme (Fig. 1, shown in gray).

* Standard error of the fit.

** Note that k_CoR_ and k_E_/k_F_ for BaL, are different from [10]. This is due to the slight variability between the current series of experiments and those performed several years ago. In the previous set of measurements, all three steps of BaL fusion (CD4 binding, CCR5 binding and escape from C52L) occurred nearly simultaneously, thus accounting for undefined (immeasurably large) k_CoR_ and k_E_/k_F_.

**Table 2:** Effects of HNP-1 on anti-HIV activity of antibodies and peptide inhibitors.

Antibody/Peptide		IC_50_ (HXB2)*		Fold decrease	IC_50_ (BaL)*		Fold decrease
		Control	+HNP-1		Control	+HNP-1	
**gp120**	17b	N.I.	N.I.	–	N.I.	N.I.	–
	scFv m9	3.71±0.06**	4.2±0.7	0.9	13.2±0.8	15.7±0.5	0.8
	m36	0.48±0.02	0.38±0.01	1.3	3.66±0.06	2.8±0.3	1.3
	m18	6.8±0.6	3.9±0.4	1.7	N.I.	N.I.	–
	PG9	N.I.	N.I.	–	19±2	17±1	1.1
	PG16	N.I	N.I	–	65±4	61±6	1.1
**gp41 N-HR**	bF-3674	4.8±0.3	0.31±0.02	15.6‴	31±2	1.08±0.05	28.7‴
	D5	107±2	3.8±0.3	28.2‴	178±8	8.9±0.3	20.0‴
	8k8	6.8±0.3	0.99±0.03	6.9‴	58±2	2.5±0.2	23.2‴
	C34	6.6±0.9	0.6±0.1	11.0‴	27±5	1.3±0.1	20.8‴
	N36^mut(e,g)^	131±12	5.5±0.4	23.8‴	N.I.	2.4±0.2	∞
	NC-1	N.I.†	N.I.†	–	N.I.	N.I.	–
**gp41 cluster I and cluster II** ¶	50-69	N.I.	N.I.	–	N.I.	N.I.	–
	240-D	N.I.	N.I.	–	N.I.	N.I.	–
	5-helix	31±2	9±1	3.4‴	208±25	64±5	3.3″
	98-6	N.I.	N.I.	–	N.I.	N.I.	–
	167-D IV	N.I.	N.I.	–	N.I.	N.I.	–
**gp41 MPER**	2F5	2.2±0.1	0.97±0.04	2.3‴	N.I.	5.0±0.1	∞
	4E10	8.1±0.8	3.6±0.2	2.3″	N.I.	11.5±0.7	∞

* The IC_50_ values for antibodies are in µg/ml, for N36^mut(e,g)^ in µM, for 5-helix and C34 in nM.

** Standard error of the fit. Statistical analysis of IC_50_ data obtained by non-linear fitting was based on the sum of squares reduction test (″ denotes P<0.0003 and ‴ denotes P<0.0001).

N.I., no inhibition within the concentration range tested.

† A slight fusion-enhancing effect.

∞ signifies a dramatic decrease in the IC_50_ from an infinitely large to a measureable value.

N.D., not determined.

¶ Antibodies to the gp41 disulfide-linked loop (cluster I [60]) and the gp41 C-HR domain (including cluster II [60]); MPER, gp41 membrane-proximal extracellular region.

We next examined the effect of HNP-1 on the kinetics of pseudovirus fusion. Experiments were carried out in medium containing 7.3 µM HNP-1 and 10% human serum. This concentration of defensin caused modest (15–20%) reduction of the HXB2 fusion (Fig. 1B, Inset), whereas the BaL fusion was not significantly diminished (P>0.35). We found that under these conditions defensin slowed down the HXB2 Env binding to CXCR4, but not to CD4. In contrast, defensin did not significantly alter the rates of CD4 or CCR5 binding by BaL (Fig. 1 and Table 1). Importantly, both HXB2 and BaL acquired resistance to C52L at much slower rates than in control experiments. These results show that defensin delays the HIV-1 entry process in the presence of serum while marginally affecting the extent of fusion.

As discussed above, the slower post-CD4 binding steps of fusion must extend the lifetime of PHIs on the cell surface. Using our kinetic model, we calculated that HNP-1 prolonged the PHI exposure on HXB2 and BaL pseudoviruses by 2- and 5-fold, respectively. Delayed CXCR4 binding and slower endocytosis/fusion equally contributed to the increased PHI lifetime for HXB2 pseudoviruses, whereas delayed steps downstream of coreceptor binding were solely responsible for the prolonged exposure of BaL intermediates in defensin-treated samples (Fig. 1 and Table 1). Delayed HIV-1 fusion was also observed when experiments were carried out in the absence of serum using a lower concentration of defensin to achieve partial inhibition of fusion (Fig. S2A, B). Also, in control experiments, the linearized HNP-1 analogue, Abu-HNP, did not affect the kinetics of HIV-1 fusion (Fig. S2C).

**Figure 2:**
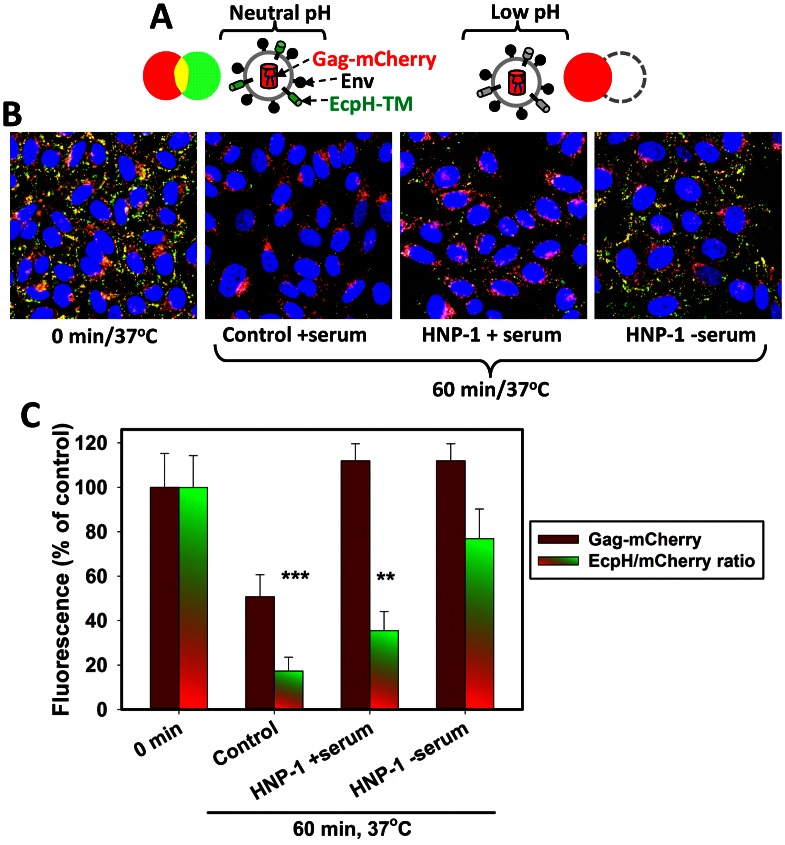
HNP-1 prevents HIV-1 detachment and interferes with virus endocytosis. (A) A diagram of pseudoviruses co-labeled with Ecliptic pHluorin fused to the ICAM-1 transmembrane domain (EcpH-TM, green) and Gag-mCherry (red) at neutral and low pH. (B) HIV-1 endocytosis assay using pseudoviruses co-labeled with EcpH-TM/Gag-mCherry. HXB2 pseudoviruses were pre-bound to TZM-bl entry in the cold (first panel), and virus entry was allowed to occur by incubating for 1 h at 37°C in the absence (second panel) or in the presence of 8.7 µM HNP-1 (third panel) or 20 µM HNP-1 in HBSS/10% serum (fourth panel). (C) Quantification of the effect of HNP-1 on uptake and dissociation of HXB2 pseudoviruses pre-bound to TZM-bl cells in the cold. The total amount of cell-associated viruses was assessed based on the Gag-mCherry signal from all particles in the image field (dark red bars). The drop in the overall mCherry signal following the incubation in the absence of defensin (second dark red bar) is primarily due to virus dissociation from cells, as described in [40], [86]. HNP-1 prevented the loss of the virus from the cell surface, both in the presence and in the absence of serum. Virus entry into acidic endosomes resulted in quenching of the EcpH signal and reduction of the EcpH/mCherry (green/red) fluorescence ratio (green-red gradient bars). Data points are means and SEM from 5–11 image fields each containing 30–40 cells. ***, P<0.001, **, P<0.003 (two-tailed t-test).

To verify the conclusion that HNP-1 interferes with pseudovirus endocytosis, we measured the HIV-1 uptake using a ratiometric assay developed by our group [29], [39], [40]. Internalization of pseudoviruses co-labeled with the pH-sensitive membrane marker, EcpH-ICAM, and the pH-resistant marker for the viral core, Gag-mCherry (Fig. 2A), permitted the visualization of HIV-1 delivery into acidic endosomes. Acidification of endosomal pH resulted in quenching of the EcpH signal and thus reduction of the ratio of EcpH (green) and mCherry (red) signals (Fig. 2B, C) compared to the ratio immediately after the low temperature binding step (0 min). Incubation at 37°C resulted in virtual disappearance of green fluorescence, whereas the red signal corresponding to the viral core accumulated at the perinuclear regions (Fig. 2B, second panel). By comparison, although HIV-1 endocytosis (quenching of the EcpH signal) was not blocked by HNP-1 in the presence of serum, the mCherry signal from viral cores was rather evenly distributed throughout the cytoplasm (Fig. 2B, third panel). This phenotype, along with a somewhat greater EcpH/mCherry ratio than in control experiments (Fig. 2C), indicates that the virus uptake and/or trafficking were delayed by HNP-1, consistent with our kinetic data (Fig. 1). In agreement with our previous study [29], [39], [40], defensin strongly inhibited pseudovirus endocytosis in the absence of serum, as evidenced by a marginal reduction in the green/red fluorescence ratio (Fig. 2B and C).

To conclude, the fusion kinetics (Fig. 1) and virus imaging (Fig. 2) data are consistent with the notion that defensin prolongs the exposure of PHIs on the cell surface by slowing down HIV-1 endocytosis. However, as we have alluded above and in [10], the estimated PHI lifetime is independent of whether these intermediates are cleared by endocytosis or through fusion with the plasma membrane. In addition, defensin extends the longevity of PHIs on HXB2 pseudoviruses by slowing down the CXCR4 binding step through down regulation of CXCR4 expression and/or by direct competition with Env-CXCR4 binding [29]. Since the lifetime of gp41 intermediates correlates with the HIV-1 sensitivity to peptides derived from the C-HR domain [10], we sought to determine whether HNP-1 could potentiate anti-HIV activity of fusion inhibitors and neutralizing antibodies. Toward this goal, we compared the sensitivity of HXB2 and BaL pseudoviruses to a panel of conformation-specific antibodies against distinct epitopes on gp120 and gp41 in the presence and in the absence of HNP-1.

### HNP-1 does not sensitize HIV-1 to anti-gp120 antibodies

**Figure 3:**
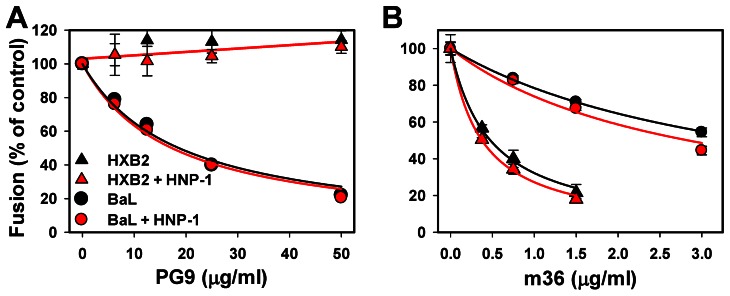
Neutralizing activity of anti-gp120 antibodies is not affected by HNP-1. Varied concentrations of neutralizing antibodies against distinct domains of HIV-1 gp120, PG9 (A), and m36 (B), were added to TZM-bl cells decorated with HXB2 (triangles) or BaL (circles) pseudoviruses immediately prior to raising the temperature to 37°C for 90 min. Experiments were performed either in absence (black symbols) or in the presence (red symbols) of 7.3 µM HNP-1 in HBSS supplemented with 10% human serum. Virus-cell fusion was measured by the BlaM assay. Data points are means and SEM from a representative triplicate experiment (PG9) or form two duplicate experiments (m36). Solid curves are obtained by non-linear curve fit to F = 100/(1+[X]/IC_50_), where [X] is the concentration of an inhibitor or an antibody. See Table 2 for the respective IC_50_ values. The experimental points showing no detectable reduction in the fusion signal were fitted with a straight line.

The neutralizing activities of several anti-gp120 antibodies were assessed using the synchronized fusion protocol whereupon viruses were pre-bound to cells at 4°C, and their entry/fusion were initiated by raising the temperature in the presence of varied concentrations of antibodies in a serum-containing medium. Since HIV-1 attached to cells in the cold remains sensitive to CD4 binding inhibitors (Fig. 1 and [8], [10], [41]), this protocol can be used to evaluate the ability of antibodies to interfere with the post-attachment steps of fusion upstream of receptor binding. First, we examined antibodies against conformational gp120 epitopes exposed in the context of the native Env trimer. Broadly neutralizing PG9 and PG16 antibodies against a quaternary glycan-containing epitope on the trimeric gp120 [42]–[45] inhibited BaL, but not HXB2 fusion at concentrations up to 50 µg/ml (Fig. 3A, Fig. S3A and Table 2). By comparison, the m18 mAb which recognizes the gp120 CD4 binding site [46] did not interfere with the BaL fusion within the concentration range tested, but partially inhibited HXB2 fusion (Fig. S3B and Table 2). Significantly, neither of these antibodies was rendered more potent by a sub-inhibitory dose of HNP-1 in the presence of serum (Table 2).

We next determined whether HNP-1 could modulate the activity of mAbs to CD4-induced gp120 epitopes: 17b [47], [48], scFv m9 (single-chain variable fragment) and m36 [21], [49], which recognize the gp120 epitopes overlapping with or adjacent to the coreceptor binding site [49], [50]. The inhibitory activity of 17b against either HXB2 or BaL was not detectable, while scFvm9 and m36 significantly reduced fusion of both HIV-1 strains (Fig. 3B, Fig. S3C and Table 2). As observed for other anti-gp120 antibodies, a sub-inhibitory dose of HNP-1 did not enhance the HIV-1 neutralization by antibodies against CD4-induced epitopes (Table 2).

### Sub-inhibitory doses of HNP-1 enhance the activity of inhibitory peptides and antibodies targeting the gp41 coiled coil domain

**Figure 4:**
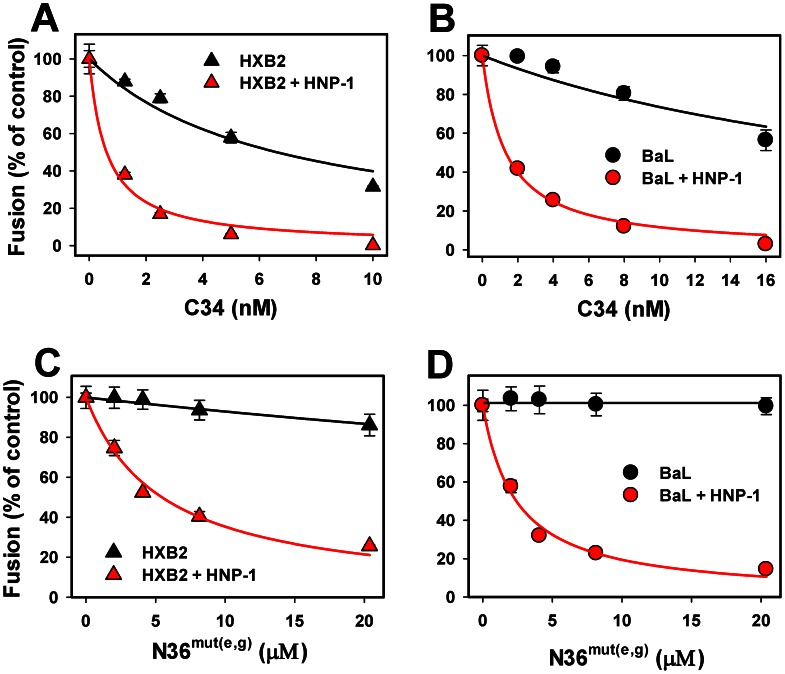
HNP-1 potentiates HIV-1 inhibition by peptides targeting the gp41 N-HR domain. Fusion experiments between HXB2 (A, C) and BaL (B, D) pseudoviruses and TZM-bl cells were performed as described in the legend to Figure 2, using varied concentrations of C34 (A, B) or N36^mut(e,g)^ (C, D) in the presence (red symbols) or in absence (black symbols) of 7.3 µM HNP-1 in HBSS supplemented with 10% human serum. Data points are means and SEM from a representative triplicate experiment; the solid lines are obtained by curve-fitting, as described above. See Table 2 for the respective IC_50_ values.

We assessed the effect of HNP-1 on inhibition of HIV-cell fusion by C34 and N36^mut(e, g)^ peptides targeting the complementary N-HR (coiled coil) domain [4], [51]. The C-HR-derived C34 peptide blocks HIV-1 fusion by binding to the complementary N-HR region and preventing the formation of 6HB. The HXB2 and BaL fusion was much more potently inhibited by C34 when a sub-inhibitory concentration of HNP-1 was present in a serum-containing medium (Fig. 4A, B and Table 2). The potentiation of C34 activity by defensin was particularly apparent for BaL pseudoviruses, which were somewhat more resistant to C34 than HXB2. Since the potency of C-HR-derived peptides correlates with the lifetime of gp41 PHIs [10], this result supports our conclusion based on the kinetics data that defensin prolongs the N-HR exposure on the cell surface.

The original N36 peptide derived from the N-HR is thought to prevent the 6HB formation by binding to the complementary C-HR region of gp41 [4]. However, the mutant N36^mut(e, g)^ peptide, in which several non-conservative substitutions of hydrophobic residues at the heptad repeat positions *e* and *g* were made, inhibits HIV-1 fusion in spite of its inability to bind the C-HR domain [51]. This result implies that N36^mut(e, g)^ interferes with gp41-mediated fusion by forming non-functional heterotrimers with the N-HR segments [51], [52]. Dose-response experiments showed that N36^mut(e, g)^ only marginally reduced the extent of HXB2 fusion and did not affect the BaL fusion at concentrations up to 20 µM (Fig. 4C, D). A sub-inhibitory dose of HNP-1 in the presence of serum dramatically enhanced the inhibitory activity of the mutant peptide. Given the low baseline sensitivity of BaL to N36^mut(e, g)^, the enhancing effect of HNP-1 was particularly striking. Defensin reduced the IC_50_ for this peptide from extremely large (undefined) to 2.4 µM (Table 2).

Next, we tested whether HNP-1 can enhance the activity of mAbs to the N-HR domain, D5, 8K8 and the bivalent Fab 3674 (bF-3674). D5 binds to the hydrophobic pocket within the gp41 coiled coil and thus interferes with the C-HR binding and 6HB formation [53], [54]. The binding site of 8K8 partially overlaps with the D5 binding site, but the latter antibody has been reported to exhibit a greater specificity for the unoccupied N-HR domain than D5 [24], [55]. bF-3674 recognizes the shallow groove on the coiled coil domain, which in the 6HB structure is located between the two C-HR segment bound to the major hydrophobic grooves of the coiled coil [56]. This antibody is therefore expected to bind to both the coiled coil and 6HB structures. The synergy between bF-3674- and C34-mediated block of HIV-1 entry [56] supports the notion that these inhibitors bind to non-overlapping sites on gp41.

**Figure 5:**
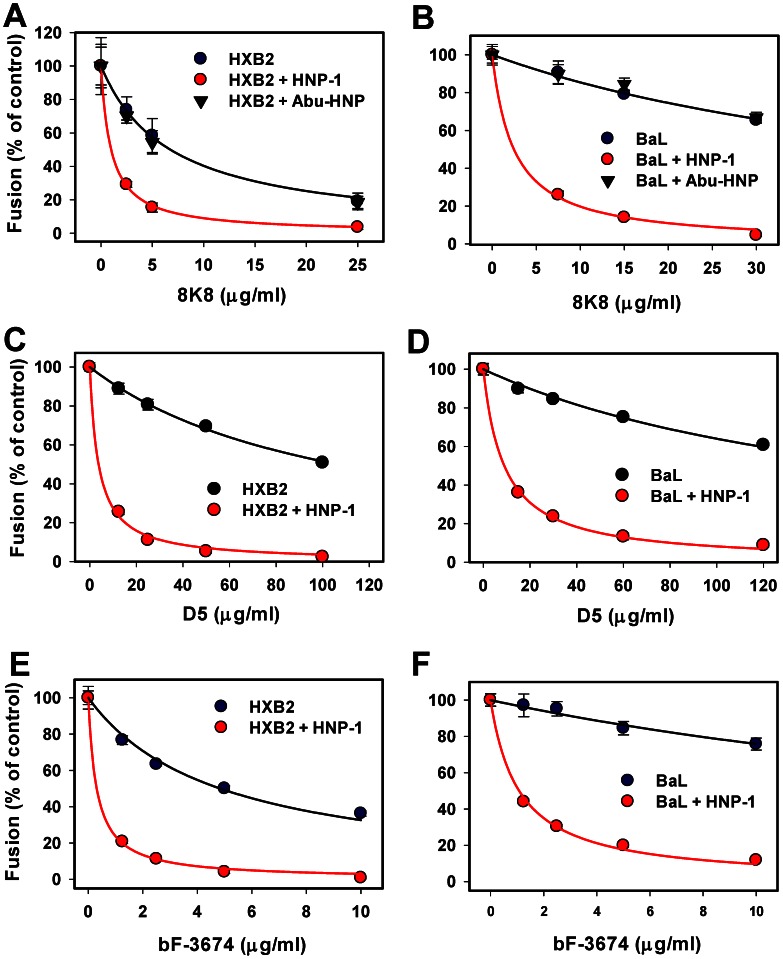
Neutralizing activities of antibodies to the gp41 N-HR domain are potentiated by α-defensin. TZM-bl cells were allowed to bind HXB2 (A, C, E) or BaL (B, D, F) pseudoviruses in the cold, overlaid with medium containing neutralizing mAbs, 8k8 (A, B), D5 (C, D) or bF-3674 (E, F) in the absence (black symbols) or in the presence (red symbols) of 7.3 µM HNP-1. See Table 2 for the respective IC_50_ values. In control experiments, titration with 8k8 was done in the presence of 7.5 µM of the inactive linearized HNP-1 mutant Abu-HNP (inverted black triangles). Data points are means and SEM from one (bF-3674 and 8k8/BaL) or two (D5 and 8k8 for HXB2) experiments performed in triplicate.

While all three antibodies inhibited HXB2 and BaL fusion, 8K8 and bF-3674 were considerably more potent in our assay than D5 (Fig. 5). Overall, BaL was more resistant to these antibodies than HXB2, except that the less potent D5 inhibited both viruses with nearly equal efficiency. A low dose of HNP-1 in the presence of serum greatly enhanced the anti-HIV activity of all three antibodies compared to control experiments (Fig. 5 and Table 2). As with the inhibitory peptides targeting the N-HR domain, the enhancing effect of defensin on BaL neutralization by mAbs was more apparent than for HXB2. We also found that a sub-inhibitory dose of HNP-1 retains its ability to sensitize HIV-1 to neutralizing antibodies in media with higher (25%) serum content (Fig. S4). In control experiments, the inactive linear analog of HNP-1 (Abu-HNP) lacking the critical disulfide bonds did not have any effect on the inhibitory activity of the 8k8 antibody (Fig. 5A, B).

We also tested whether defensin could confer neutralizing activity to a non-neutralizing monoclonal antibody NC-1, which recognizes both the free N-HR and the N-HR in the context of 6HBs [52], [57]. Within the concentration range tested, NC-1 did not inhibit HXB2 or BaL fusion, either in the presence or absence of HNP-1 (Table 2). The marked enhancement of the anti-HIV activity of peptides and neutralizing antibodies targeting the N-HR domain implies that this region is normally not exposed for a sufficiently long time to allow optimal binding of these inhibitors.

### HNP-1 augments the activity of peptides and antibodies targeting the gp41 C-HR and MPER

To test the effect of HNP-1 on inhibitors targeting the gp41 C-HR domain, we used the 5-helix peptide [58]. This peptide consists of a single polypeptide chain in which three N-HR segments are interspersed with two C-HR segments, leaving a vacant groove that avidly binds C-HR-derived peptides [12]. The HIV-1 fusion experiments in the presence of escalating doses of 5-helix revealed that HXB2 was much more sensitive to this peptide than BaL (Fig. S5). A sub-inhibitory dose of HNP-1 in the presence of serum modestly (∼3-fold) reduced the IC_50_ of 5-helix against either viral strain (Fig. S5 and Table 2).

We next tested the synergy between HNP-1 and non-neutralizing or weakly neutralizing antibodies against the gp41 regions downstream of the N-HR, using “cluster I” antibodies against the disulfide-linked loop and “cluster II” antibodies against the C-HR domain. Cluster I mAbs, 50-69 [59] and 240-D [60], bind to the monomeric or oligomeric gp41 loop region, whereas cluster II antibodies, 167-D IV and 98-6 [60], appear to bind to the post-fusion 6HB structure [61]–[65]. Neither cluster I nor cluster II antibodies exhibited detectable neutralizing activity under our experimental conditions (Table 2). As was the case with NC-1 antibody against the N-HR domain, defensin did not confer the HIV-neutralizing ability to the gp41 cluster I or cluster II mAbs.

**Figure 6:**
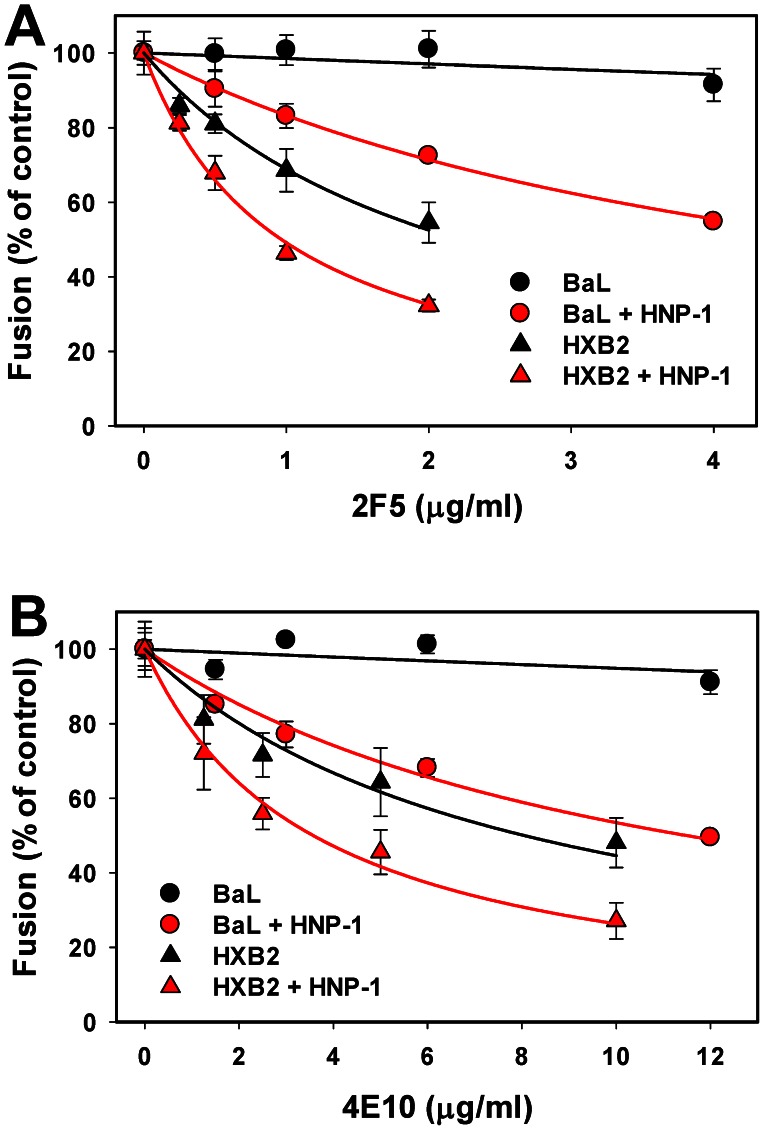
HNP-1 enhances neutralizing activity of antibodies against the membrane-proximal extracellular domain of gp41. Inhibition of HXB2 (triangles) and BaL (circles) pseudotype fusion with TZM-bl cells by 2F5 (A) and 4E10 (B) mAbs in the presence (red symbols) or in the absence (black symbols) of 7.3 µM HNP-1 in HBSS supplemented with 10% human serum. Data points are means and SEM from a representative experiment performed in triplicate (see Table 2 for IC_50_ values).

Next, we examined the interactions between HNP-1 and broadly neutralizing antibodies against the gp41 membrane-proximal extracellular region (MPER), 2F5 [66] and 4E10 [67]. These antibodies recognize the native Env relatively poorly, but exhibit improved binding to CD4-induced conformations of gp41 [22], [68]–[71]. In our experimental system, 2F5 and 4E10 inhibited HXB2 fusion but were less efficient against BaL within the concentration range tested (Fig. 6). Defensin modestly reduced the IC_50_ for both antibodies against HXB2 pseudoviruses. By contrast, compared to the virtual lack of inhibition of BaL fusion by 2F5 and 4E10, HNP-1 caused dramatic potentiation of their neutralizing activity (Fig. 6 and Table 2).

### Anti-gp41 antibodies and patient serum more effectively neutralize HIV-1 entry into PBMCs in the presence of α-defensin

**Figure 7:**
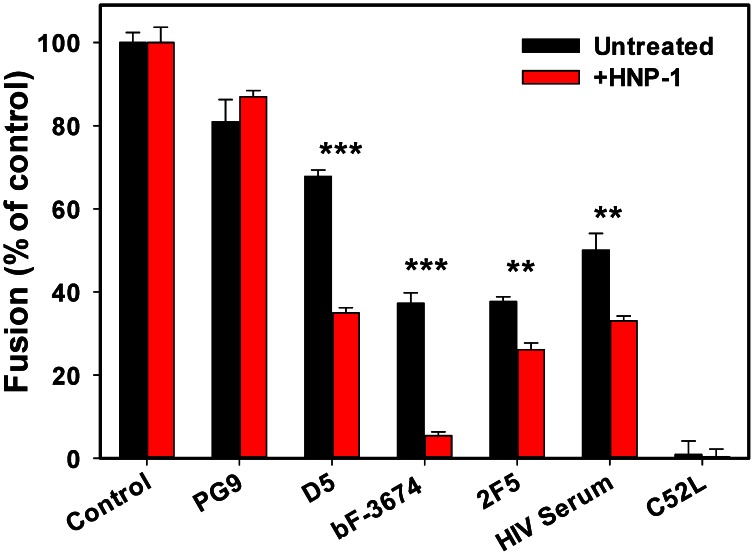
HNP-1 synergizes with neutralizing antibodies to gp41 and with HIV serum in inhibiting HIV-1 fusion. HXB2 pseudoviruses were pre-bound to human PBMCs in the cold, and fusion was initiated in the absence or in the presence of indicated antibodies by shifting to 37°C. Data are normalized to the extent of fusion (measured by the BlaM assay) in the absence of defensin or antibodies (control). Addition of 24 µM HNP-1 in HBSS with 10% human serum did not affect HXB2 fusion. The following concentrations of antibodies were used: D5 (25 µg/ml), bF-3674 (12 µg/ml), 2F5 (2.5 µg/ml), PG9 (20 µg/ml). HIV-1 neutralizing serum was diluted 1∶15 prior to adding to cells. Experiments were performed either in the absence (black bars) or in the presence (red bars) of 24 µM HNP-1 in 10% human serum. Experiment with 3 µM C52L were used as a negative control. Data points are means and SEM from two independent experiments (D5, bF-3674 and HIV serum) or a representative experiment (PG9 and 2F5) performed in triplicate. **, P<0.005; ***, P<0.001 based on the two-tailed t-test.

To test our conclusion that a sub-inhibitory dose of HNP-1 strongly potentiates the activity of antibodies against gp41 pre-hairpins in a more physiologically-relevant system, we examined HIV-1 fusion with PBMCs. PBMCs were adhered to a poly-lysine coated 96-well plate and allowed to bind pseudoviruses at 4°C, as described in Materials and Methods. Viruses and cells were incubated with or without antibodies in the presence or absence of defensin at 37°C for 90 min to allow fusion. To evaluate the effect of defensin on HIV-1 neutralization by PG9, D5, bF-3674 and 2F5 in these target cells, we compared the fusion efficiencies after incubation with fixed concentrations of antibodies close to their respective IC_50_s (when possible) with and without HNP-1 in serum-containing medium. Notably, whereas 24 µM of HNP-1 did not have a detectable effect on HXB2 fusion with PBMCs (Fig. 7), defensin enhanced the activity of all tested anti-gp41 antibodies. The fusion inhibitory activity of bF-3674 was particularly strongly enhanced by HNP-1. As was observed with TZM-bl cells (Fig. 3A), the anti-gp120 antibody PG9 marginally attenuated the extent of HXB2 fusion with PBMCs, and this effect was not modulated by HNP-1.

We next asked whether α-defensin could augment the neutralizing activity of human HIV serum. PBMCs were centrifuged with HXB2 pseudoviruses in the cold, washed and incubated at 37°C with or without pooled serum from AIDS patients, either in the presence or in the absence of defensin. HIV serum diminished the HXB2 fusion with PBMCs by ∼50%, whereas the combination of immune serum with a non-inhibitory dose of HNP-1 further decreased the fusion signal to 33% of the control (Fig. 7). Synergy between HNP-1 and HIV immune serum reveals an important beneficial role of α-defensin in enhancing the neutralizing activity of naturally occurring human antibodies.

## Discussion

We have previously adapted a direct virus-cell fusion assay to dissect the early steps of HIV-1 entry and have developed a kinetic model to evaluate the lifetime of gp41 pre-hairpin intermediates [10]. Here, we applied these methodologies to investigate the effect of sub-inhibitory concentrations of α-defensin on the kinetics of HIV-1 fusion and its sensitivity to inhibition/neutralization by antiviral peptides and antibodies. Defensin extended the exposure of gp41 intermediates by slowing down HIV-1 uptake/fusion. In addition, HNP-1 delayed the CXCR4 binding step of HXB2 entry. The prolonged PHI exposure correlated with enhanced anti-viral activity of fusion inhibitors and antibodies targeting the transiently exposed gp41 domains, with the most profound effect observed with the N-HR-targeting inhibitors. The neutralization-enhancing effect of HNP-1 was confirmed using PBMCs which are the natural targets for HIV infection.

The observed relationship between longevity of PHIs and the efficacy of fusion inhibitors and antibodies supports the kinetic restriction on HIV-1 neutralization. Thus, in addition to steric factors blocking the antibody access to respective epitopes, HIV-1 appears to kinetically limit the antibody or peptide binding to gp41 by minimizing the exposure of key transitional epitopes. While the kinetic effect of defensin appears to be the most likely explanation for HIV-1 sensitization, we cannot rule out the possibility that HNP-1 increases the accessibility of conserved epitopes by creating a more “open” Env conformation. However, the fact that the smaller C-HR-derived peptides, whose binding to the gp41 N-HR is not sterically restricted, are also potentiated by defensin, does not support the “open” conformation model. Of note, the kinetic and steric restriction may not be mutually exclusive, since the prolonged PHI lifetimes could augment the antibody binding by allowing more time for transient exposure of poorly accessible epitopes.

Although the mechanism by which HNP-1 prolongs the PHI lifetime is not understood, our previous study has demonstrated the ability of this defensin to (i) slow down the CXCR4 binding, (ii) interfere with the 6HB formation, apparently through interactions with the gp41 HR domains, and (iii) inhibit HIV-1 uptake [29]. The longevity of gp41 PHIs, which can form upon CD4 engagement, is determined by the rates of coreceptor binding and productive endocytosis or fusion with the plasma membrane, as depicted in Figure 1. While delayed folding into 6HBs (k_F_) stabilizes PHIs, the rate of productive endocytosis (k_E_) or, in the alternative model, the rate of fusion (k_F_) ultimately determines the availability of fusion intermediates on the cell surface. It thus appears that defensin prolongs the PHI exposure by delaying the CXCR4 binding (for HXB2 pseudoviruses) and, more universally, by slowing down the fusion steps downstream of coreceptor binding.

It is possible that the observed lack of HNP-1 effect on anti-gp120 mAbs and the overall modest neutralizing activity of these antibodies resulted from the post-attachment neutralization protocol employed in this study. We chose a post-attachment neutralization assay in order to separate the HIV-1 binding and fusion steps and to minimize the effect of defensin-mediated down regulation of CD4 and CXCR4 expression [29]. Our protocol thus reduces the complexity of the HNP-1 effects on HIV-1 fusion and enables the kinetic measurements of this process. Importantly, HIV-1 Env does not appear to irreversibly engage CD4 after a brief pre-binding step at 4°C, as evidenced by the ability of BMS-806 and anti-CD4 antibodies to fully block HIV-1 fusion (Fig. 1 and [8], [10], [17]). These findings show that our experimental protocol is suitable for studies of HIV-1 neutralization by anti-gp120 mAbs. We therefore surmise that the undetectable effect of defensin on anti-gp120 antibodies is due to the lack of kinetic control over their binding to respective epitopes and probably the lack of significant competition for binding to these epitopes.

We found that HNP-1 selectively stimulated the anti-viral activity of peptides and antibodies against the N-HR domain, whereas potentiation of inhibitors targeting other gp41 domains was relatively modest (Table 2). The reason for this selective effect is presently unclear. The C-HR domains, for example, appear to be at least partially occluded on the native Env [72]. Kinetic studies of the 5-helix binding and inhibition of gp41-mediated fusion indicate that C-HR may be exposed for only a few seconds [12]. It is therefore reasonable to expect that stabilization of PHIs would result in a stronger reduction of the IC_50_ for 5-helix (Table 2). However, we have previously shown that the activity of C34, but not of 5-helix, is enhanced upon stabilizing the gp41 fusion intermediates [73]. This result suggests that the 5-helix binding sites may not be exposed throughout the lifetime of PHIs. It is thus possible that the modest decrease in the IC_50_ for 5-helix in the presence of defensin reflects its weak effect on the C-HR exposure.

Our results with 2F5 and 4E10 in the presence of defensin and serum are indicative of different degrees of the MPER occlusion on Env from different isolates. The dramatic enhancement of the 2F5 and 4E10 activity against BaL pseudoviruses, which are otherwise relatively resistant to these antibodies, suggests that HNP-1 markedly prolongs the availability of their respective epitopes. The less pronounced effect of defensin on HXB2 neutralization by these mAbs, on the other hand, is consistent with considerable exposure of MPER of this protein prior to or during fusion. These findings are in agreement with the report that the MPER domains are exposed on the native Env from lab-adapted strains (HXB2, ADA), but occluded on BaL and on Env glycoproteins from neutralization-resistant primary isolates [74].

Another explanation for the modest enhancing effect of defensin on antibodies and peptides targeting the gp41 domains other than the N-HR is that HNP-1 may inhibit the 6HB formation [29] by binding to the gp41 C-HR domain in a manner similar to retrocyclin (a θ-defensin) [75]. This could result in competition between HNP-1 and mAbs and/or peptides for binding to the C-HR domain, as has been reported for antibodies to the gp120 CD4-binding and coreceptor-binding sites [30]. However, it appears unlikely that sub-inhibitory doses of defensin in the presence of serum could significantly reduce the antibody binding to their epitopes. The fact that defensin does not attenuate HIV-1 neutralization by any of the antibodies used in this study (Table 2) supports this notion. While defensin could also bind to N-HR, this possibility appears inconsistent with its strong enhancing effect on peptides and antibodies targeting this region.

The enhanced HIV neutralization by immune serum in the presence of HNP-1 implies that the prolonged exposure of PHIs is also beneficial for antibodies circulating in the bloodstream. Of note, the level of human α-defensin in plasma can reach 6.5 µM [76], [77], a concentration that is close to one used in our study. The enhancing effect of HNP-1 on anti-gp41 antibodies is not without precedent. Sub-inhibitory doses of N36^mut(e, g)^ have been reported to synergize with bF-3674, 2F5 and 4E10, apparently by sequestering the N-HR domains and thereby extending the temporal window for antibody binding [14].

In conclusion, this study reveals the previously unappreciated role of innate immunity peptides in enhancing adaptive immune responses to HIV-1 infection. This finding suggests new strategies to improve therapeutic regimens and vaccine efforts. Specifically, our data and published results [14] demonstrate the utility of developing small molecule compounds capable of stabilizing intermediate conformations of HIV-1 Env *in vivo* and thereby potentiating the neutralizing activity of antibodies against this glycoprotein.

## Materials and Methods

### Cells and reagents

HeLa-derived indicator TZM-bl cells expressing CD4, CXCR4, and CCR5 were grown in DMEM supplemented with 10% FBS (HyClone Laboratories, Logan, UT) and penicillin/streptomycin (Sigma, St. Louis, MO). HEK 293T/17 cells (ATCC, Manassas, VA) were grown in the same medium supplemented with 0.5 mg/ml geneticin (Invitrogen, Grand Island, NY). Human peripheral blood mononuclear cells (PBMCs) were isolated from whole blood and activated with 10 ng/ml IL-2 and 2.5 µg/ml phytohemagglutinin (PHA, Sigma), as described previously [17]. All media and buffers were obtained from HyClone (Thermo Fisher Scientific, Logan, UT) or Cellgro (Mediatech Inc., Manassas, VA). Human serum was obtained from Atlanta Biologicals (Lawrenceville, GA).

The following cell lines and reagents were obtained from the NIH AIDS Research and Reference Reagent Program: IL-2 (from Dr. M. Gately, Hoffmann-La Roche) [78], indicator TZM-bl cells (from Drs. J. Kappes and X. Wu) [79], HIV-1 immune serum (Dr. L. Vujcic, FDA) [80], HIV monoclonal antibodies (mAbs) PG9 and PG16 (from IAVI, La Jolla, CA) [42], 17b (Dr. J. Robinson, Tulane University Medical Center) [47], [48], 2F5 and 4E10 (Dr. Hermann Katinger, University of Natural Resources, Vienna, Austria) [66], [67], 50-69, 98-6, 240-D and 167-D IV (Dr. S. Zolla-Pazner, Veterans Administration Medical Center, New York) [59], [60], NC-1 (Dr. S. Jiang, New York Blood Center, NY) [57], TAK-779 (Division of AIDS, NIAID) and pcDNA3.1 vector expressing HIV-1 BaL Env (clone BaL.01, Dr. J. Mascola, NIH) [81].

The antibodies scFv m9, m36, m18 were a gift from Dr. D. Dimitrov (NCI, Frederick, MD), the 8K8 mAb was provided by Dr. M. Zwick (Scripps Research Institute, CA), the bivalent Fab 3674 (bF-3674) was from Dr. M. Clore (NIDDK, NIH), and the D5 mAb was from Dr. M. Miller (Merck). The pCAGGS plasmid encoding HXB2 Env was provided by Dr. J. Binley (Torrey Pines Institute). The HIV-1-based packaging vector pR8ΔEnv lacking the *env* gene was from Dr. D. Trono (Geneva, Switzerland). The C52L recombinant peptide was a gift from Dr. Min Lu (New Jersey Medical School) [38]. The C34 peptide was a gift from Dr. L. Wang (Institute of Human Virology, University of Maryland Baltimore), and 5-Helix was a gift from Dr. M. Root (Thomas Jefferson University). BMS-806 was purchased from ChemPacific Corp. (Baltimore, MD), and AMD3100 was from Sigma.

### Synthesis and purification of HNP-1 and its derivatives

HNP-1 and its linear analog Abu-HNP-1, in which the six Cys residues are replaced by the isosteric α-aminobutyric acid, as well as the N36^mut(e, g)^ peptide were all prepared *via* Boc solid phase peptide synthesis using an optimized coupling chemistry developed by Kent and colleagues [82]. Oxidative refolding of HNP-1 was performed as described [83], and structural validation of synthetic HNP1 was achieved by X-ray crystallography [84]. All peptides were purified to homogeneity by reversed phase HPLC and their molecular masses ascertained by electrospray ionization mass spectrometry. Peptide concentrations were quantified by UV absorbance measurements at 280 nm using molar extinction coefficients calculated by a published algorithm [85].

### Virus production and characterization

Pseudoviruses were produced by transfection of 293T cells using PolyFect reagent (QIAGEN, Valencia, CA), as described in [17]. Briefly, for the BlaM assay, cells were transfected with a mixture of the following plasmids: 2 µg of pR8ΔEnv, 2 µg of BlaM-Vpr-expressing pMM310 vector, 1 µg of pcRev plasmid, and 3 µg of vectors encoding HIV-1 BaL or HXB2 Env. Transfection media was replaced with phenol red-free media after an overnight incubation, and cell culture medium was collected at 48 h post-transfection. Virus-containing medium was passed through a 0.45 µm filter, aliquoted, and stored at −80°C. For experiments with PBMCs, viruses were concentrated by pelleting onto a 20% sucrose cushion or using Lenti-X concentrator (Clontech Laboratories, CA). The infectious titer of the virus stock was determined using TZM-bl cells, as described previously [15].

### Virus-cell fusion assay

Unless otherwise stated, all HIV-cell fusion experiments were done in the presence of 10% human serum. HIV-1 pseudovirus fusion with target cells was measured using the BlaM assay, as described previously [15], [17]. HXB2 or BaL pseudoviruses with β-lactamase-Vpr (BlaM-Vpr) chimera incorporated into the viral core were bound to PBMCs or TZM-bl cells by centrifugation at 4°C for 30 min at 1,550×*g*. For fusion experiments with PBMCs, cells were allowed to adhere to a poly-L-lysine coated 96-well plate (2•10^5^ cells/well) in Hanks' buffer (HBSS) for 30 min at room temperature. Excess cells were removed and wells were blocked with 10% FBS-supplemented HBSS for 15 min. HXB2 pseudoviruses (4•10^5^ IU/well) were pre-bound to adhered PBMCs by spinoculation, as described above. After the virus binding step, cells were washed once with HBSS and incubated at 37°C for 90 min to allow virus entry. The fusion reaction was stopped by placing the plates on ice, and the culture medium was replaced with the BlaM substrate, CCF4-AM (Invitrogen). Cells were left at 12°C overnight, and the BlaM activity was determined from the ratio of blue and green fluorescence signals, using the Synergy HT fluorescence plate reader (Bio-Tek Instruments, Germany).

The effect of HNP-1 on the kinetics of HIV-1 fusion with TZM-bl cells was assessed by the “time of addition” experiments, as described in [10]. Briefly, viruses were pre-bound to cells by centrifugation in the cold, and the virus entry was initiated by raising the temperature to 37°C. Fusion was stopped at indicated time points by adding fully inhibitory concentrations of inhibitors. The rate of CD4 binding was determined by adding BMS-806 (20 µM), CXCR4 and CCR5 binding was assessed using AMD3100 (7 µM) and TAK-779 (30 µM), respectively. The rate of receptor/coreceptor-mediated endocytosis was measured by adding C52L (1 µM), which blocks the gp41 6-helix bundle formation. At the end of the incubation (37°C, 90 min), fusion was stopped by chilling the cells, and the BlaM activity was measured.

### HIV-1 endocytosis

Internalization of pseudoviruses by TZM-bl cells was measured, as previously described [29], [39], [40]. Briefly, HXB2 pseudoparticles were co-labeled with the pH-sensitive derivative of GFP, ecliptic pHlourin (EcpH), fused to the N-terminus of the ICAM-1 transmembrane domain (EcpH-ICAM) and with the HIV-1 Gag-mCherry chimera, as the viral core marker. Although Gag-mCherry is not cleaved by the HIV-1 protease [29], [39], [40], inclusion of wild-type HIV-1 pR8ΔEnv vector in the transfection mixture yielded pseudoviruses capable of single-round infection. Quenching of EcpH-ICAM upon entry into early endosomes enables the measurements of HIV-1 uptake and delivery into mildly acidic compartments expressed as the ratio of the EcpH-ICAM signal to the pH-resistant mCherry signal.

### HNP-1 effect on inhibitory activity of neutralizing antibodies and peptide inhibitors

Pseudoviruses were pre-bound to cells by spinoculation in the cold and the excess virus was removed by washing with HBSS. Varied doses of neutralizing antibodies or inhibitory peptides (C34, N36^mut(e,g)^ or 5-Helix) were added to cells in HBSS/10% human serum in the absence or in the presence of 7.3 µM of HNP-1. Fusion was immediately initiated by shifting cells to 37°C. After 90 min, cells were placed on ice to stop fusion, loaded with the CCF4-AM substrate, and incubated overnight at 12°C. The resulting BlaM signal was measured, as described above.

## Supporting Information

Figure S1 **Dose-dependence of HNP-1 inhibition of HXB2 pseudotype fusion with TZM-bl cells in the presence and in the absence of human serum.** HXB2 pseudoviruses were pre-bound to TZM-bl cells in the cold and allowed to enter and fuse by incubating at 37°C for 90 min, either in the absence (open triangles) or in the presence (filled triangles) of 10% human serum. Data points are means and SEM from a representative triplicate experiment. The vertical dashed line corresponds to 7.3 µM HNP-1 used in this study to assess the effects of defensin in the presence of serum.
(TIFF)

Figure S2 **Sub-inhibitory doses of HNP-1 slow down HIV-1 fusion in the absence of serum.** HXB2 pseudoviruses were pre-bound to TZM-bl cells in the cold and allowed to undergo fusion for 90 min at 37°C, either in the absence (A) or in the presence (B) of 1.2 µM HNP-1 in HBSS without serum. (C) Fusion experiments were performed in the presence of 7.5 µM of the linearized HNP-1 mutant (Abu-HNP) in serum-containing medium. Fusion was stopped at indicated time points by adding fully inhibitory concentrations of BMS-806, AMD3100 or C52L, and the resulting virus fusion was measured by the BlaM assay. Data points are means and SEM from a representative experiment performed in triplicate.
(TIFF)

Figure S3 **Neutralizing activity of anti-gp120 antibodies in the presence of HNP-1.** TZM-bl cells were allowed to bind HXB2 (triangles) or BaL (circles) pseudoviruses in the cold, and fusion was initiated by incubation at 37°C for 90 min in the presence of escalating doses of neutralizing antibodies, PG16 (A), m18 (B), scFv m9 (C). Experiments were performed either in absence (black symbols) or in the presence (red symbols) of 7.3 µM HNP-1 in HBSS/10% human serum, and the resulting fusion was measured by the BlaM assay. Data points are means and SEM from a representative triplicate experiment; the scFv m9 data are form two triplicate experiments. Solid curves are obtained by non-linear curve fit to F = 100/(1+[X]/IC_50_), where [X] is the concentration of an inhibitor or an antibody (see Table 2 for the respective IC_50_ values). The experimental points showing no detectable reduction in the fusion signal were fit to a straight line.
(TIFF)

Figure S4 **HNP-1 retains the ability to potentiate neutralizing activity of D5 antibody in medium with high serum content.** HXB2 pseudoviruses were pre-bound to TZM-bl cells at 4°C and shifted to 37°C for 90 min to initiate fusion, as measured by the BlaM assay. (A) HXB2 fusion experiments were carried out in the presence of varied doses of HNP-1 in media containing 0, 25 or 50% of human serum in HBSS. Based on these results, a sub-inhibitory concentration of 10 µM HNP-1, which inhibited HXB2 fusion in 25 and 50% serum by ∼15–20%, was selected for the virus neutralization experiments. (B) The effect of 10 µM HNP-1 on HIV-1 neutralization by the D5 monoclonal antibody. Viruses and cells were exposed to escalating doses of D5 in HBSS that was supplemented with 25% human serum in the presence or in the absence of defensin.
(TIFF)

Figure S5 **Potentiation of the 5-helix activity by HNP-1.** HXB2 (A) and BaL (B) fusion with TZM-bl cells was carried out by adding different concentrations of 5-helix, either in the presence (red symbols) or in the absence (black symbols) of HNP-1 (7.3 µM) in 10% human serum. Data points are means and SEM from a representative triplicate experiment (see Table 2 for IC_50_ values).
(TIFF)

## References

[1] Wyatt R, Sodroski J (1998) The HIV-1 envelope glycoproteins: fusogens, antigens, and immunogens. Science 280: 1884–1888. 9632381 10.1126/science.280.5371.1884

[2] Berger EA, Murphy PM, Farber JM (1999) Chemokine receptors as HIV-1 coreceptors: roles in viral entry, tropism, and disease. Annu Rev Immunol 17: 657–700. 10358771 10.1146/annurev.immunol.17.1.657

[3] Doms RW (2000) Beyond receptor expression: the influence of receptor conformation, density, and affinity in HIV-1 infection. Virology 276: 229–237. 11040114 10.1006/viro.2000.0612

[4] Eckert DM, Kim PS (2001) Mechanisms of Viral Membrane Fusion and Its Inhibition. Annu Rev Biochem 70: 777–810. 11395423 10.1146/annurev.biochem.70.1.777

[5] Melikyan GB (2008) Common principles and intermediates of viral protein-mediated fusion: the HIV-1 paradigm. Retrovirology 5: 111. 19077194 10.1186/1742-4690-5-111 PMC2633019

[6] Blumenthal R, Durell S, Viard M (2012) HIV viral entry and envelope glycoprotein mediated fusion. J Biol Chem 287: 40841–9. 23043104 10.1074/jbc.R112.406272 PMC3510787

[7] Furuta RA, Wild CT, Weng Y, Weiss CD (1998) Capture of an early fusion-active conformation of HIV-1 gp41. Nat Struct Biol 5: 276–279. 9546217 10.1038/nsb0498-276

[8] Melikyan GB, Markosyan RM, Hemmati H, Delmedico MK, Lambert DM, et al (2000) Evidence that the transition of HIV-1 gp41 into a six-helix bundle, not the bundle configuration, induces membrane fusion. J Cell Biol 151: 413–424. 11038187 10.1083/jcb.151.2.413 PMC2192659

[9] Kilgore NR, Salzwedel K, Reddick M, Allaway GP, Wild CT (2003) Direct evidence that C-peptide inhibitors of human immunodeficiency virus type 1 entry bind to the gp41 N-helical domain in receptor-activated viral envelope. J Virol 77: 7669–7672. 12805467 10.1128/JVI.77.13.7669-7672.2003 PMC164814

[10] Miyauchi K, Kozlov MM, Melikyan GB (2009) Early steps of HIV-1 fusion define the sensitivity to inhibitory peptides that block 6-helix bundle formation. PLoS Pathog 5: e1000585. 19763181 10.1371/journal.ppat.1000585 PMC2736578

[11] Gallo SA, Reeves JD, Garg H, Foley B, Doms RW, et al (2006) Kinetic studies of HIV-1 and HIV-2 envelope glycoprotein-mediated fusion. Retrovirology 3: 90. 17144914 10.1186/1742-4690-3-90 PMC1693918

[12] Steger HK, Root MJ (2006) Kinetic dependence to HIV-1 entry inhibition. J Biol Chem 281: 25813–25821. 16803885 10.1074/jbc.M601457200

[13] Reeves JD, Gallo SA, Ahmad N, Miamidian JL, Harvey PE, et al (2002) Sensitivity of HIV-1 to entry inhibitors correlates with envelope/coreceptor affinity, receptor density, and fusion kinetics. Proc Natl Acad Sci U S A 99: 16249–16254. 12444251 10.1073/pnas.252469399 PMC138597

[14] Gustchina E, Bewley CA, Clore GM (2008) Sequestering of the prehairpin intermediate of gp41 by peptide N36Mut(e,g) potentiates the human immunodeficiency virus type 1 neutralizing activity of monoclonal antibodies directed against the N-terminal helical repeat of gp41. J Virol 82: 10032–10041. 18667502 10.1128/JVI.01050-08 PMC2566276

[15] Miyauchi K, Kim Y, Latinovic O, Morozov V, Melikyan GB (2009) HIV enters cells via endocytosis and dynamin-dependent fusion with endosomes. Cell 137: 433–444. 19410541 10.1016/j.cell.2009.02.046 PMC2696170

[16] Markosyan RM, Cohen FS, Melikyan GB (2003) HIV-1 envelope proteins complete their folding into six-helix bundles immediately after fusion pore formation. Mol Biol Cell 14: 926–938. 12631714 10.1091/mbc.E02-09-0573 PMC151570

[17] de la Vega M, Marin M, Kondo N, Miyauchi K, Kim Y, et al (2011) Inhibition of HIV-1 endocytosis allows lipid mixing at the plasma membrane, but not complete fusion. Retrovirology 8: 99. 22145853 10.1186/1742-4690-8-99 PMC3297528

[18] Labrijn AF, Poignard P, Raja A, Zwick MB, Delgado K, et al (2003) Access of antibody molecules to the conserved coreceptor binding site on glycoprotein gp120 is sterically restricted on primary human immunodeficiency virus type 1. J Virol 77: 10557–10565. 12970440 10.1128/JVI.77.19.10557-10565.2003 PMC228502

[19] Hamburger AE, Kim S, Welch BD, Kay MS (2005) Steric accessibility of the HIV-1 gp41 N-trimer region. J Biol Chem 280: 12567–12572. 15657041 10.1074/jbc.M412770200

[20] Eckert DM, Shi Y, Kim S, Welch BD, Kang E, et al (2008) Characterization of the steric defense of the HIV-1 gp41 N-trimer region. Protein Sci 17: 2091–2100. 18802030 10.1110/ps.038273.108 PMC2590922

[21] Chen W, Zhu Z, Feng Y, Dimitrov DS (2008) Human domain antibodies to conserved sterically restricted regions on gp120 as exceptionally potent cross-reactive HIV-1 neutralizers. Proc Natl Acad Sci U S A 105: 17121–17126. 18957538 10.1073/pnas.0805297105 PMC2579388

[22] Frey G, Peng H, Rits-Volloch S, Morelli M, Cheng Y, et al (2008) A fusion-intermediate state of HIV-1 gp41 targeted by broadly neutralizing antibodies. Proc Natl Acad Sci U S A 105: 3739–3744. 18322015 10.1073/pnas.0800255105 PMC2268799

[23] Peachman KK, Wieczorek L, Polonis VR, Alving CR, Rao M (2010) The effect of sCD4 on the binding and accessibility of HIV-1 gp41 MPER epitopes to human monoclonal antibodies. Virology 408: 213–223. 20961591 10.1016/j.virol.2010.09.029

[24] Luftig MA, Mattu M, Di Giovine P, Geleziunas R, Hrin R, et al (2006) Structural basis for HIV-1 neutralization by a gp41 fusion intermediate-directed antibody. Nat Struct Mol Biol 13: 740–747. 16862157 10.1038/nsmb1127

[25] Steckbeck JD, Orlov I, Chow A, Grieser H, Miller K, et al (2005) Kinetic rates of antibody binding correlate with neutralization sensitivity of variant simian immunodeficiency virus strains. J Virol 79: 12311–12320. 16160158 10.1128/JVI.79.19.12311-12320.2005 PMC1211559

[26] Haim H, Steiner I, Panet A (2007) Time frames for neutralization during the human immunodeficiency virus type 1 entry phase, as monitored in synchronously infected cell cultures. J Virol 81: 3525–3534. 17251303 10.1128/JVI.02293-06 PMC1866073

[27] Ketas TJ, Kuhmann SE, Palmer A, Zurita J, He W, et al (2007) Cell surface expression of CCR5 and other host factors influence the inhibition of HIV-1 infection of human lymphocytes by CCR5 ligands. Virology 364: 281–290. 17428518 10.1016/j.virol.2007.02.022 PMC2151978

[28] Choudhry V, Zhang MY, Harris I, Sidorov IA, Vu B, et al (2006) Increased efficacy of HIV-1 neutralization by antibodies at low CCR5 surface concentration. Biochem Biophys Res Commun 348: 1107–1115. 16904645 10.1016/j.bbrc.2006.07.163 PMC2268024

[29] Demirkhanyan LH, Marin M, Padilla-Parra S, Zhan C, Miyauchi K, et al (2012) Multifaceted mechanisms of HIV-1 entry inhibition by human alpha-defensin. J Biol Chem 287: 28821–38. 22733823 10.1074/jbc.M112.375949 PMC3436536

[30] Furci L, Sironi F, Tolazzi M, Vassena L, Lusso P (2007) Alpha-defensins block the early steps of HIV-1 infection: interference with the binding of gp120 to CD4. Blood 109: 2928–2935. 17132727 10.1182/blood-2006-05-024489

[31] Chang TL, Vargas J Jr, DelPortillo A, Klotman ME (2005) Dual role of alpha-defensin-1 in anti-HIV-1 innate immunity. J Clin Invest 115: 765–773. 15719067 10.1172/JCI200521948 PMC548697

[32] Leikina E, Delanoe-Ayari H, Melikov K, Cho MS, Chen A, et al (2005) Carbohydrate-binding molecules inhibit viral fusion and entry by crosslinking membrane glycoproteins. Nat Immunol 6: 995–1001. 16155572 10.1038/ni1248

[33] Gallo SA, Puri A, Blumenthal R (2001) HIV-1 gp41 six-helix bundle formation occurs rapidly after the engagement of gp120 by CXCR4 in the HIV-1 Env-mediated fusion process. Biochemistry 40: 12231–12236. 11591141 10.1021/bi0155596

[34] Lin PF, Blair W, Wang T, Spicer T, Guo Q, et al (2003) A small molecule HIV-1 inhibitor that targets the HIV-1 envelope and inhibits CD4 receptor binding. Proc Natl Acad Sci U S A 100: 11013–11018. 12930892 10.1073/pnas.1832214100 PMC196918

[35] Si Z, Madani N, Cox JM, Chruma JJ, Klein JC, et al (2004) Small-molecule inhibitors of HIV-1 entry block receptor-induced conformational changes in the viral envelope glycoproteins. Proc Natl Acad Sci U S A 101: 5036–5041. 15051887 10.1073/pnas.0307953101 PMC387369

[36] Donzella GA, Schols D, Lin SW, Este JA, Nagashima KA, et al (1998) AMD3100, a small molecule inhibitor of HIV-1 entry via the CXCR4 co-receptor. Nat Med 4: 72–77. 9427609 10.1038/nm0198-072

[37] Baba M, Nishimura O, Kanzaki N, Okamoto M, Sawada H, et al (1999) A small-molecule, nonpeptide CCR5 antagonist with highly potent and selective anti-HIV-1 activity. Proc Natl Acad Sci U S A 96: 5698–5703. 10318947 10.1073/pnas.96.10.5698 PMC21923

[38] Deng Y, Zheng Q, Ketas TJ, Moore JP, Lu M (2007) Protein design of a bacterially expressed HIV-1 gp41 fusion inhibitor. Biochemistry 46: 4360–4369. 17371053 10.1021/bi7001289

[39] Jha NK, Latinovic O, Martin E, Novitskiy G, Marin M, et al (2011) Imaging single retrovirus entry through alternative receptor isoforms and intermediates of virus-endosome fusion. PLoS Pathog 7: e1001260. 21283788 10.1371/journal.ppat.1001260 PMC3024281

[40] Miyauchi K, Marin M, Melikyan GB (2011) Visualization of retrovirus uptake and delivery into acidic endosomes. Biochem J 434: 559–569. 21175427 10.1042/BJ20101588 PMC3249399

[41] Henderson HI, Hope TJ (2006) The temperature arrested intermediate of virus-cell fusion is a functional step in HIV infection. Virol J 3: 36. 16725045 10.1186/1743-422X-3-36 PMC1482684

[42] Walker LM, Phogat SK, Chan-Hui PY, Wagner D, Phung P, et al (2009) Broad and potent neutralizing antibodies from an African donor reveal a new HIV-1 vaccine target. Science 326: 285–289. 19729618 10.1126/science.1178746 PMC3335270

[43] Pancera M, McLellan JS, Wu X, Zhu J, Changela A, et al (2010) Crystal structure of PG16 and chimeric dissection with somatically related PG9: structure-function analysis of two quaternary-specific antibodies that effectively neutralize HIV-1. J Virol 84: 8098–8110. 20538861 10.1128/JVI.00966-10 PMC2916520

[44] McLellan JS, Pancera M, Carrico C, Gorman J, Julien JP, et al (2011) Structure of HIV-1 gp120 V1/V2 domain with broadly neutralizing antibody PG9. Nature 480: 336–343. 22113616 10.1038/nature10696 PMC3406929

[45] Davenport TM, Friend D, Ellingson K, Xu H, Caldwell Z, et al (2011) Binding interactions between soluble HIV envelope glycoproteins and quaternary-structure-specific monoclonal antibodies PG9 and PG16. J Virol 85: 7095–7107. 21543501 10.1128/JVI.00411-11 PMC3126577

[46] Prabakaran P, Gan J, Wu YQ, Zhang MY, Dimitrov DS, et al (2006) Structural mimicry of CD4 by a cross-reactive HIV-1 neutralizing antibody with CDR-H2 and H3 containing unique motifs. J Mol Biol 357: 82–99. 16426633 10.1016/j.jmb.2005.12.062

[47] Sullivan N, Sun Y, Sattentau Q, Thali M, Wu D, et al (1998) CD4-Induced conformational changes in the human immunodeficiency virus type 1 gp120 glycoprotein: consequences for virus entry and neutralization. J Virol 72: 4694–4703. 9573233 10.1128/jvi.72.6.4694-4703.1998 PMC109994

[48] Thali M, Moore JP, Furman C, Charles M, Ho DD, et al (1993) Characterization of conserved human immunodeficiency virus type 1 gp120 neutralization epitopes exposed upon gp120-CD4 binding. J Virol 67: 3978–3988. 7685405 10.1128/jvi.67.7.3978-3988.1993 PMC237765

[49] Zhang MY, Shu Y, Rudolph D, Prabakaran P, Labrijn AF, et al (2004) Improved breadth and potency of an HIV-1-neutralizing human single-chain antibody by random mutagenesis and sequential antigen panning. J Mol Biol 335: 209–219. 14659751 10.1016/j.jmb.2003.09.055

[50] Kwong PD, Wyatt R, Robinson J, Sweet RW, Sodroski J, et al (1998) Structure of an HIV gp120 envelope glycoprotein in complex with the CD4 receptor and a neutralizing human antibody [see comments]. Nature 393: 648–659. 9641677 10.1038/31405 PMC5629912

[51] Bewley CA, Louis JM, Ghirlando R, Clore GM (2002) Design of a novel peptide inhibitor of HIV fusion that disrupts the internal trimeric coiled-coil of gp41. J Biol Chem 277: 14238–14245. 11859089 10.1074/jbc.M201453200

[52] Dimitrov AS, Louis JM, Bewley CA, Clore GM, Blumenthal R (2005) Conformational changes in HIV-1 gp41 in the course of HIV-1 envelope glycoprotein-mediated fusion and inactivation. Biochemistry 44: 12471–12479. 16156659 10.1021/bi051092d PMC1314968

[53] Miller MD, Geleziunas R, Bianchi E, Lennard S, Hrin R, et al (2005) A human monoclonal antibody neutralizes diverse HIV-1 isolates by binding a critical gp41 epitope. Proc Natl Acad Sci U S A 102: 14759–14764. 16203977 10.1073/pnas.0506927102 PMC1253587

[54] Sabin C, Corti D, Buzon V, Seaman MS, Lutje Hulsik D, et al (2010) Crystal structure and size-dependent neutralization properties of HK20, a human monoclonal antibody binding to the highly conserved heptad repeat 1 of gp41. PLoS Pathog 6: e1001195. 21124990 10.1371/journal.ppat.1001195 PMC2987821

[55] Nelson JD, Kinkead H, Brunel FM, Leaman D, Jensen R, et al (2008) Antibody elicited against the gp41 N-heptad repeat (NHR) coiled-coil can neutralize HIV-1 with modest potency but non-neutralizing antibodies also bind to NHR mimetics. Virology 377: 170–183. 18499210 10.1016/j.virol.2008.04.005 PMC2493441

[56] Gustchina E, Louis JM, Lam SN, Bewley CA, Clore GM (2007) A monoclonal Fab derived from a human nonimmune phage library reveals a new epitope on gp41 and neutralizes diverse human immunodeficiency virus type 1 strains. J Virol 81: 12946–12953. 17898046 10.1128/JVI.01260-07 PMC2169134

[57] Jiang S, Lin K, Lu M (1998) A conformation-specific monoclonal antibody reacting with fusion-active gp41 from the human immunodeficiency virus type 1 envelope glycoprotein. J Virol 72: 10213–10217. 9811763 10.1128/jvi.72.12.10213-10217.1998 PMC110570

[58] Root MJ, Kay MS, Kim PS (2001) Protein design of an HIV-1 entry inhibitor. Science 291: 884–888. 11229405 10.1126/science.1057453

[59] Gorny MK, Gianakakos V, Sharpe S, Zolla-Pazner S (1989) Generation of human monoclonal antibodies to human immunodeficiency virus. Proc Natl Acad Sci U S A 86: 1624–1628. 2922401 10.1073/pnas.86.5.1624 PMC286751

[60] Xu JY, Gorny MK, Palker T, Karwowska S, Zolla-Pazner S (1991) Epitope mapping of two immunodominant domains of gp41, the transmembrane protein of human immunodeficiency virus type 1, using ten human monoclonal antibodies. J Virol 65: 4832–4838. 1714520 10.1128/jvi.65.9.4832-4838.1991 PMC248941

[61] Golding H, Zaitseva M, de Rosny E, King LR, Manischewitz J, et al (2002) Dissection of human immunodeficiency virus type 1 entry with neutralizing antibodies to gp41 fusion intermediates. J Virol 76: 6780–6790. 12050391 10.1128/JVI.76.13.6780-6790.2002 PMC136262

[62] Gorny MK, Zolla-Pazner S (2000) Recognition by human monoclonal antibodies of free and complexed peptides representing the prefusogenic and fusogenic forms of human immunodeficiency virus type 1 gp41. J Virol 74: 6186–6192. 10846104 10.1128/jvi.74.13.6186-6192.2000 PMC112119

[63] Yuan W, Li X, Kasterka M, Gorny MK, Zolla-Pazner S, et al (2009) Oligomer-specific conformations of the human immunodeficiency virus (HIV-1) gp41 envelope glycoprotein ectodomain recognized by human monoclonal antibodies. AIDS Res Hum Retroviruses 25: 319–328. 19292593 10.1089/aid.2008.0213 PMC2853836

[64] Frey G, Chen J, Rits-Volloch S, Freeman MM, Zolla-Pazner S, et al (2010) Distinct conformational states of HIV-1 gp41 are recognized by neutralizing and non-neutralizing antibodies. Nat Struct Mol Biol 17: 1486–1491. 21076402 10.1038/nsmb.1950 PMC2997185

[65] Dennison SM, Anasti K, Scearce RM, Sutherland L, Parks R, et al (2011) Nonneutralizing HIV-1 gp41 envelope cluster II human monoclonal antibodies show polyreactivity for binding to phospholipids and protein autoantigens. J Virol 85: 1340–1347. 21106741 10.1128/JVI.01680-10 PMC3020517

[66] Buchacher A, Predl R, Strutzenberger K, Steinfellner W, Trkola A, et al (1994) Generation of human monoclonal antibodies against HIV-1 proteins; electrofusion and Epstein-Barr virus transformation for peripheral blood lymphocyte immortalization. AIDS Res Hum Retroviruses 10: 359–369. 7520721 10.1089/aid.1994.10.359

[67] Stiegler G, Kunert R, Purtscher M, Wolbank S, Voglauer R, et al (2001) A potent cross-clade neutralizing human monoclonal antibody against a novel epitope on gp41 of human immunodeficiency virus type 1. AIDS Res Hum Retroviruses 17: 1757–1765. 11788027 10.1089/08892220152741450

[68] Dimitrov AS, Jacobs A, Finnegan CM, Stiegler G, Katinger H, et al (2007) Exposure of the Membrane-Proximal External Region of HIV-1 gp41 in the Course of HIV-1 Envelope Glycoprotein-Mediated Fusion. Biochemistry 46: 1398–1401. 17260969 10.1021/bi062245f

[69] de Rosny E, Vassell R, Jiang S, Kunert R, Weiss CD (2004) Binding of the 2F5 monoclonal antibody to native and fusion-intermediate forms of human immunodeficiency virus type 1 gp41: implications for fusion-inducing conformational changes. J Virol 78: 2627–2631. 14963170 10.1128/JVI.78.5.2627-2631.2004 PMC369236

[70] Finnegan CM, Berg W, Lewis GK, DeVico AL (2002) Antigenic properties of the human immunodeficiency virus transmembrane glycoprotein during cell-cell fusion. J Virol 76: 12123–12134. 12414953 10.1128/JVI.76.23.12123-12134.2002 PMC136862

[71] Alam SM, Scearce RM, Parks RJ, Plonk K, Plonk SG, et al (2008) Human immunodeficiency virus type 1 gp41 antibodies that mask membrane proximal region epitopes: antibody binding kinetics, induction, and potential for regulation in acute infection. J Virol 82: 115–125. 17942537 10.1128/JVI.00927-07 PMC2224348

[72] Sattentau QJ, Moore JP (1991) Conformational changes induced in the human immunodeficiency virus envelope glycoprotein by soluble CD4 binding. J Exp Med 174: 407–415. 1713252 10.1084/jem.174.2.407 PMC2118908

[73] Abrahamyan LG, Mkrtchyan SR, Binley J, Lu M, Melikyan GB, et al (2005) The cytoplasmic tail slows the folding of human immunodeficiency virus type 1 Env from a late prebundle configuration into the six-helix bundle. J Virol 79: 106–115. 15596806 10.1128/JVI.79.1.106-115.2005 PMC538707

[74] Chakrabarti BK, Walker LM, Guenaga JF, Ghobbeh A, Poignard P, et al (2011) Direct antibody access to the HIV-1 membrane-proximal external region positively correlates with neutralization sensitivity. J Virol 85: 8217–8226. 21653673 10.1128/JVI.00756-11 PMC3147955

[75] Gallo SA, Wang W, Rawat SS, Jung G, Waring AJ, et al (2006) Theta-defensins prevent HIV-1 Env-mediated fusion by binding gp41 and blocking 6-helix bundle formation. J Biol Chem 281: 18787–18792. 16648135 10.1074/jbc.M602422200

[76] Panyutich AV, Panyutich EA, Krapivin VA, Baturevich EA, Ganz T (1993) Plasma defensin concentrations are elevated in patients with septicemia or bacterial meningitis. J Lab Clin Med 122: 202–207. 8340706

[77] Shiomi K, Nakazato M, Ihi T, Kangawa K, Matsuo H, et al (1993) Establishment of radioimmunoassay for human neutrophil peptides and their increases in plasma and neutrophil in infection. Biochem Biophys Res Commun 195: 1336–1344. 8216266 10.1006/bbrc.1993.2190

[78] Lahm HW, Stein S (1985) Characterization of recombinant human interleukin-2 with micromethods. J Chromatogr 326: 357–361. 3875623 10.1016/s0021-9673(01)87461-6

[79] Wei X, Decker JM, Liu H, Zhang Z, Arani RB, et al (2002) Emergence of resistant human immunodeficiency virus type 1 in patients receiving fusion inhibitor (T-20) monotherapy. Antimicrob Agents Chemother 46: 1896–1905. 12019106 10.1128/AAC.46.6.1896-1905.2002 PMC127242

[80] Vujcic LK, Quinnan GV Jr (1995) Preparation and characterization of human HIV type 1 neutralizing reference sera. AIDS Res Hum Retroviruses 11: 783–787. 7546904 10.1089/aid.1995.11.783

[81] Li Y, Svehla K, Mathy NL, Voss G, Mascola JR, et al (2006) Characterization of antibody responses elicited by human immunodeficiency virus type 1 primary isolate trimeric and monomeric envelope glycoproteins in selected adjuvants. J Virol 80: 1414–1426. 16415019 10.1128/JVI.80.3.1414-1426.2006 PMC1346938

[82] Schnolzer M, Alewood P, Jones A, Alewood D, Kent SB (1992) In situ neutralization in Boc-chemistry solid phase peptide synthesis. Rapid, high yield assembly of difficult sequences. Int J Pept Protein Res 40: 180–193. 1478777 10.1111/j.1399-3011.1992.tb00291.x

[83] Wu Z, Powell R, Lu W (2003) Productive folding of human neutrophil alpha-defensins in vitro without the pro-peptide. J Am Chem Soc 125: 2402–2403. 12603122 10.1021/ja0294257

[84] Wei G, de Leeuw E, Pazgier M, Yuan W, Zou G, et al (2009) Through the looking glass, mechanistic insights from enantiomeric human defensins. J Biol Chem 284: 29180–29192. 19640840 10.1074/jbc.M109.018085 PMC2781462

[85] Pace CN, Vajdos F, Fee L, Grimsley G, Gray T (1995) How to measure and predict the molar absorption coefficient of a protein. Protein Sci 4: 2411–2423. 8563639 10.1002/pro.5560041120 PMC2143013

[86] Platt EJ, Kozak SL, Durnin JP, Hope TJ, Kabat D (2010) Rapid dissociation of HIV-1 from cultured cells severely limits infectivity assays, causes the inactivation ascribed to entry inhibitors, and masks the inherently high level of infectivity of virions. J Virol 84: 3106–3110. 20042508 10.1128/JVI.01958-09 PMC2826045

