# Supplementary figures and images for: Sub-Inhibitory Concentrations of Human α-defensin Potentiate Neutralizing Antibodies against HIV-1 gp41 Pre-Hairpin Intermediates in the Presence of Serum
Source: PLoS Pathog. 2013 Jun 13;9(6):e1003431. doi: 10.1371/journal.ppat.1003431 (PMC3681749; doi:10.1371/journal.ppat.1003431)

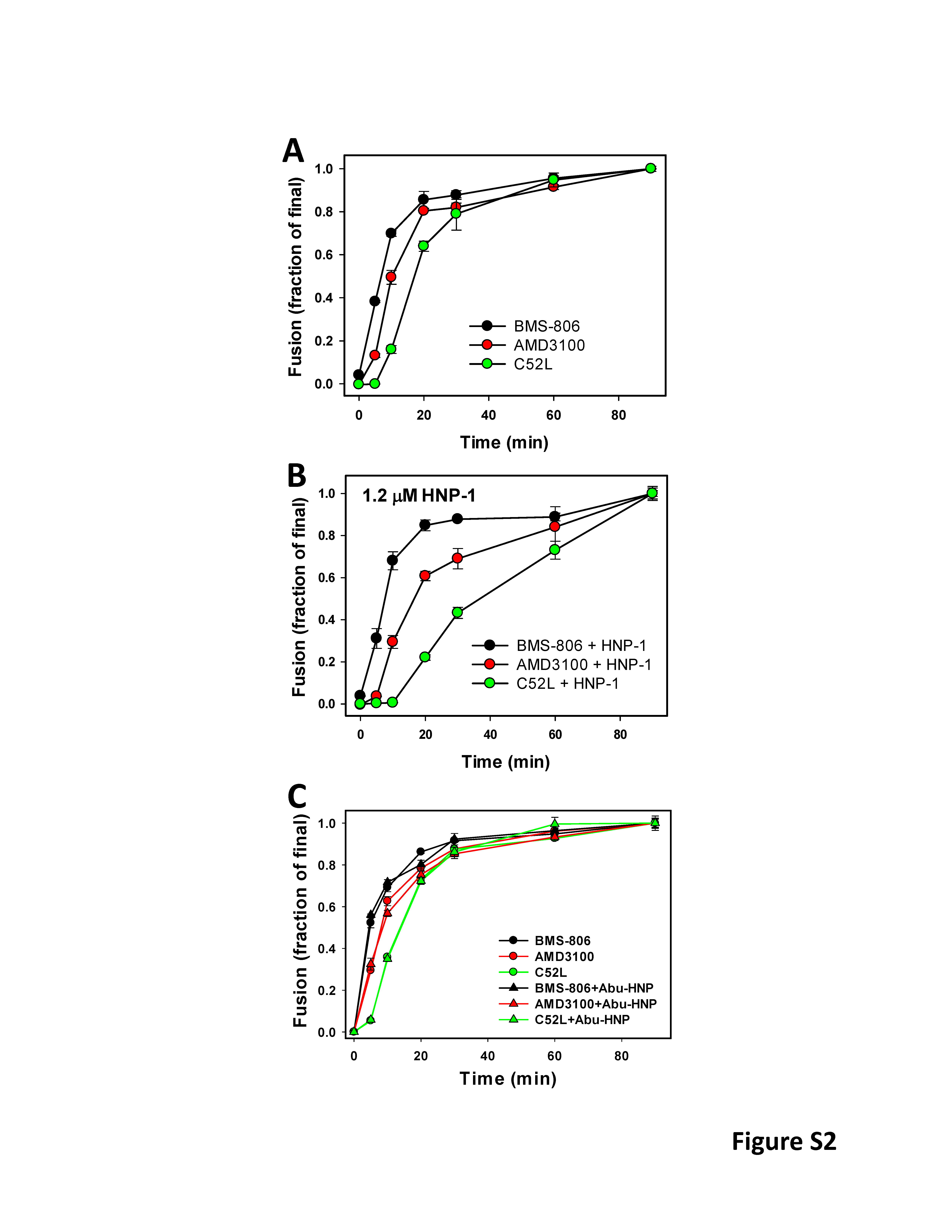

Supplement: Figure S2 — Sub-inhibitory doses of HNP-1 slow down HIV-1 fusion in the absence of serum. HXB2 pseudoviruses were pre-bound to TZM-bl cells in the cold and allowed to undergo fusion for 90 min at 37°C, either in the absence (A) or in the presence (B) of 1.2 µM HNP-1 in HBSS without serum. (C) Fusion experiments were performed in the presence of 7.5 µM of the linearized HNP-1 mutant (Abu-HNP) in serum-containing medium. Fusion was stopped at indicated time points by adding fully inhibitory concentrations of BMS-806, AMD3100 or C52L, and the resulting virus fusion was measured by the BlaM assay. Data points are means and SEM from a representative experiment performed in triplicate. (TIFF) [file ppat.1003431.s002.tiff]

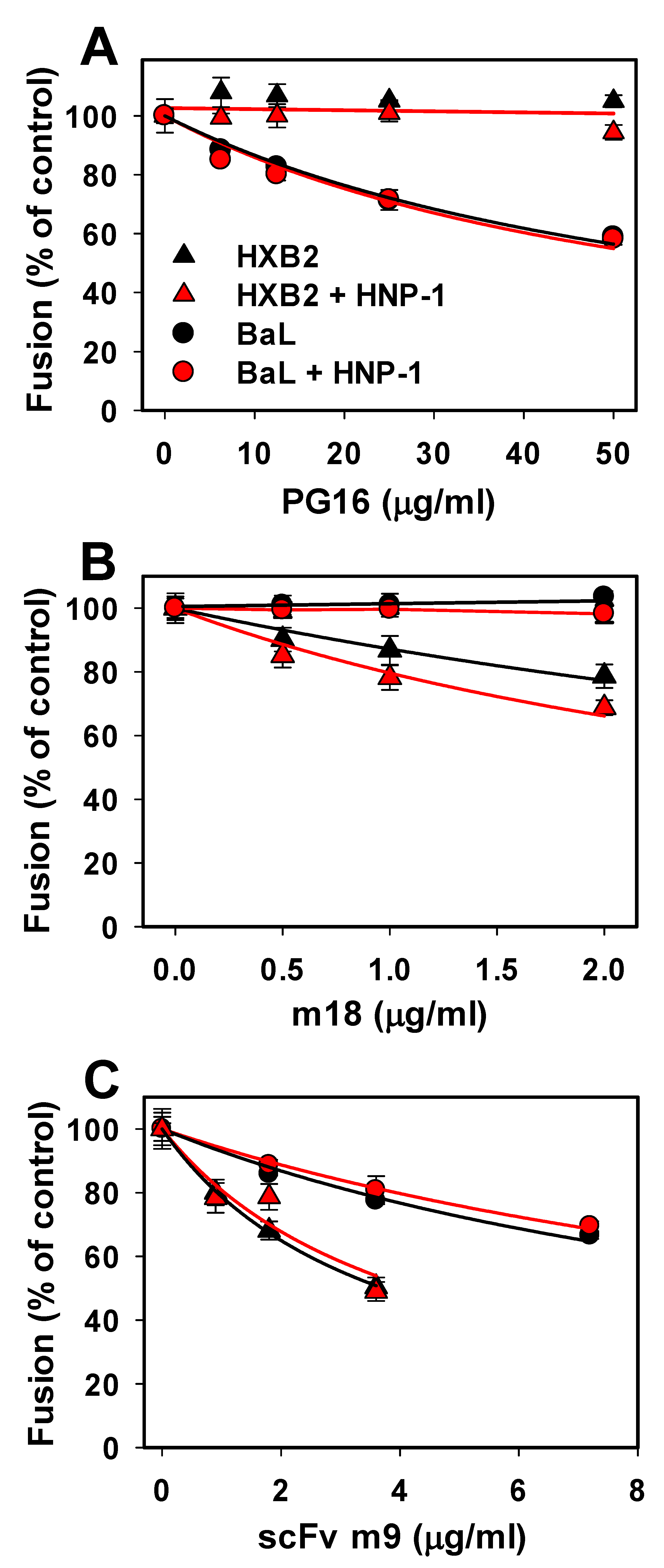

Supplement: Figure S3 — Neutralizing activity of anti-gp120 antibodies in the presence of HNP-1. TZM-bl cells were allowed to bind HXB2 (triangles) or BaL (circles) pseudoviruses in the cold, and fusion was initiated by incubation at 37°C for 90 min in the presence of escalating doses of neutralizing antibodies, PG16 (A), m18 (B), scFv m9 (C). Experiments were performed either in absence (black symbols) or in the presence (red symbols) of 7.3 µM HNP-1 in HBSS/10% human serum, and the resulting fusion was measured by the BlaM assay. Data points are means and SEM from a representative triplicate experiment; the scFv m9 data are form two triplicate experiments. Solid curves are obtained by non-linear curve fit to F = 100/(1+[X]/IC50), where [X] is the concentration of an inhibitor or an antibody (see Table 2 for the respective IC50 values). The experimental points showing no detectable reduction in the fusion signal were fit to a straight line. (TIFF) [file ppat.1003431.s003.tiff]

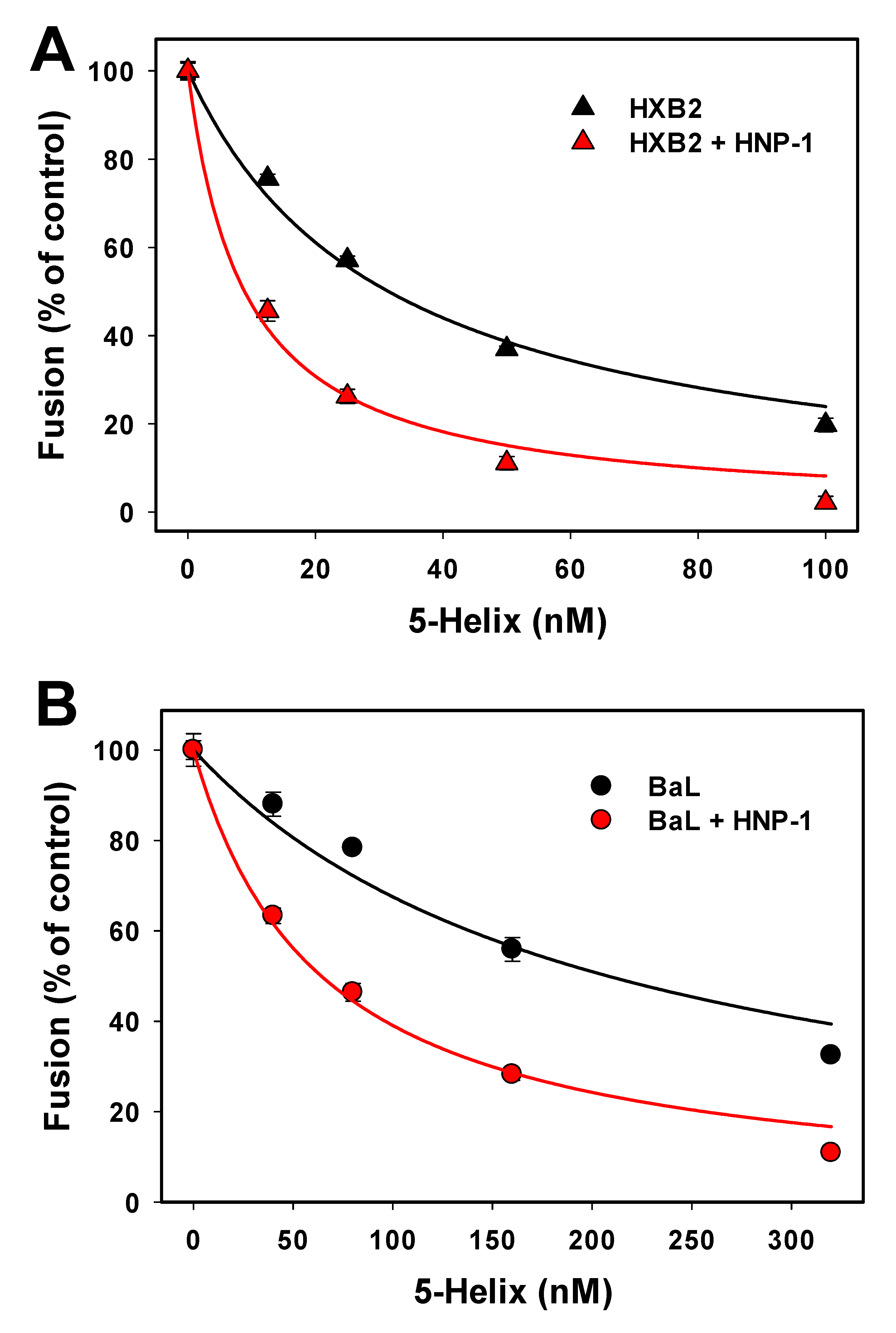

Supplement: Figure S5 — Potentiation of the 5-helix activity by HNP-1. HXB2 (A) and BaL (B) fusion with TZM-bl cells was carried out by adding different concentrations of 5-helix, either in the presence (red symbols) or in the absence (black symbols) of HNP-1 (7.3 µM) in 10% human serum. Data points are means and SEM from a representative triplicate experiment (see Table 2 for IC50 values). (TIFF) [file ppat.1003431.s005.tiff]
